# Reported health and social consequences of the COVID-19 pandemic on vulnerable populations and implemented solutions in six West African countries: A media content analysis

**DOI:** 10.1371/journal.pone.0252890

**Published:** 2021-06-16

**Authors:** Khalida Saalim, Kwame S. Sakyi, Fatema-Tuz-Zohra, Emily Morrison, Prince Owusu, Sarah L. Dalglish, Mufaro Kanyangarara

**Affiliations:** 1 Department of International Health, Johns Hopkins Bloomberg School of Public Health, Baltimore, Maryland, United States of America; 2 Department of Public and Environmental Wellness, Oakland University School of Health Sciences, Rochester, Michigan, United States of America; 3 Center for Learning and Childhood Development, Accra, Ghana; 4 Department of Epidemiology and Biostatistics, University of South Carolina Arnold School of Public Health, Columbia, South Carolina, United States of America; 5 Institute for Global Health, University College London, United Kingdom; University of Perugia, ITALY

## Abstract

Coronavirus has spread worldwide with over 140 million cases and resulting in more than 3 million deaths between November 2019 to April 2021, threatening the socio-economic and psychosocial stability of many families and communities. There has been limited research to understand the consequences of COVID-19 on vulnerable populations in West Africa, and whether such consequences differ by countries’ previous experience with Ebola. Using a media analysis of leading online news sources, this study identified the populations particularly vulnerable to the threats of the COVID-19 pandemic, described the consequences of COVID-19 experienced by these populations, and reported on the solutions to address them. All articles from the selected news sources published between January 1 and June 30, 2020 on 6 West African countries were imported into Dedoose. A total of 4,388 news articles were coded for excerpts on vulnerable populations, only 285 excerpts of which mentioned the existing effects of COVID-19 on vulnerable populations or implemented solutions. News articles from countries with past experience with Ebola were more likely to mention the pandemic’s effects on vulnerable populations, especially on incarcerated people. Vulnerable groups were reported to have experienced a range of effects including economic disruptions, heightened domestic and sexual abuse, arbitrary arrests, health care inaccessibility, and educational challenges throughout the pandemic. With implications for the achievement of the Sustainable Development Goals (SDG) for 2030 in West Africa, these countries should consider and focus more strategic efforts on vulnerable populations to overcome their fight against the COVID-19 pandemic and to achieve the SDG for 2030.

## Introduction

Coronavirus has spread worldwide with over 140 million cases and resulting in more than 3 million deaths between November 2019 to April 2021 [1]. Despite having weaker health systems than richer countries, West African countries have experienced relatively low incidence and case fatality rates [2]. The COVID-19 incidence rate in West Africa is 14 times lower than the global incidence rate and the COVID-19 case fatality rate is 1.7 times lower than globally [1, 2]. The relatively lower spread and severity of the disease in Africa have been presumably attributed to the relatively younger population with lower rates of chronic disease [3], limited testing and surveillance capacities resulting in an underestimation of the true burden of the coronavirus [4], milder symptoms in these populations despite potentially higher seroprevalence rates [5], and serological cross-reactivity against SARS-CoV-2 due to prior exposure to other coronaviruses among other explanations [6]. However, present literature further draws on the ties between social equity and COVID-19 cases and deaths. Unequitable contexts present in low-income countries, such as socioeconomic disparities and severe population density might lead to higher COVID-19 infection and death rates [7]. Alternatively, many West African countries have prior experience with the Ebola epidemic, potentially improving the pandemic response to COVID-19 [8]. However, while the direct effects of COVID-19 on mortality may appear less severe in West Africa, there have been concerning social consequences relating to the pandemic.

To reduce the spread of coronavirus, lockdowns, travel restrictions and curfews were established in some places, triggering and exacerbating economic instability and political turmoil in some African countries [9, 10]. The pandemic has also had far-reaching consequences on the psychosocial health of individuals and communities. Illness and deaths due to COVID-19, temporary and permanent layoffs, restrictions on informal income generating activities due to social distancing protocols, disruption of social celebrations and forms of entertainment, transitions to in-home learning for students, and social distancing from relatives have increased feelings of stress and anxiety for many people and interrupted the livelihoods of many populations globally including those in West Africa [11, 12].

Certain populations are more vulnerable to the negative social and economic effects of the COVID-19 pandemic due to underlying wealth inequalities, social discrimination, and social exclusion [13]. Mothers and children, prisoners, older adults, persons with disabilities, and individuals and families living in poverty are key groups who may face additional challenges in enduring the effects of the pandemic [11]. There is existing research documenting the disproportionate and varying effects of the pandemic on vulnerable populations. One study by Durizzo et. al. describes the impact of the pandemic on the mental well-being of the urban poor in African countries, including Ghana, gathered from survey results [14]. Another study in Nigeria details case reports of intimate partner violence against women in relation to economic stressors induced by the pandemic [15]. However, extensive research on the effects of the pandemic on these vulnerable populations in West Africa has been limited. Furthermore, even fewer studies have reviewed African countries’ targeted interventions to limit the effects of measures such as lockdowns on vulnerable populations and have examined the effectiveness of such interventions on the wellbeing of vulnerable populations [16, 17]. Additionally, based on our findings, not many studies have examined the role that managing the Ebola epidemic has played in a country’s response to managing COVID-19, particularly in efforts to protect vulnerable populations. The West African context provides a unique opportunity to pursue this question, as several countries in the region were the worst hit by the Ebola epidemic, potentially improving capacity to address the effects of COVID-19 on vulnerable populations [18].

The overall aim of this study was to determine the most pressing effects of the COVID-19 pandemic on vulnerable populations in West Africa using media analysis methodology. Specifically, this study sought to identify the populations notably vulnerable to the threats of COVID-19, describe the effects of the pandemic on these vulnerable populations, and catalogue the response measures used to manage such effects.

This qualitative media analysis is nested in a broader study to examine the effects of the COVID-19 pandemic on West African countries and document how the experience of the Ebola epidemic in certain countries may have influenced the management and subsequent effects of COVID-19. During the Ebola epidemic, countries established prevention measures such as lockdowns, market and school closures, similar to those experienced throughout the COVID-19 pandemic [19–21]. To address the objectives of the broader study, this study also includes a focus on how experience with Ebola may have served as lessons learned to adjust country response towards the effects of COVID-19 on vulnerable populations. We compare the effects and policy and programmatic responses to COVID-19 for vulnerable populations in six countries, four with prior experience of Ebola, and two without.

## Methods

For this study, we used an exploratory media analysis: media sources provide essential information during large-scale phenomena such as the current pandemic [22]. This media analysis follows the standards for reporting qualitative research [23].

### Country selection

**Fig 1:**
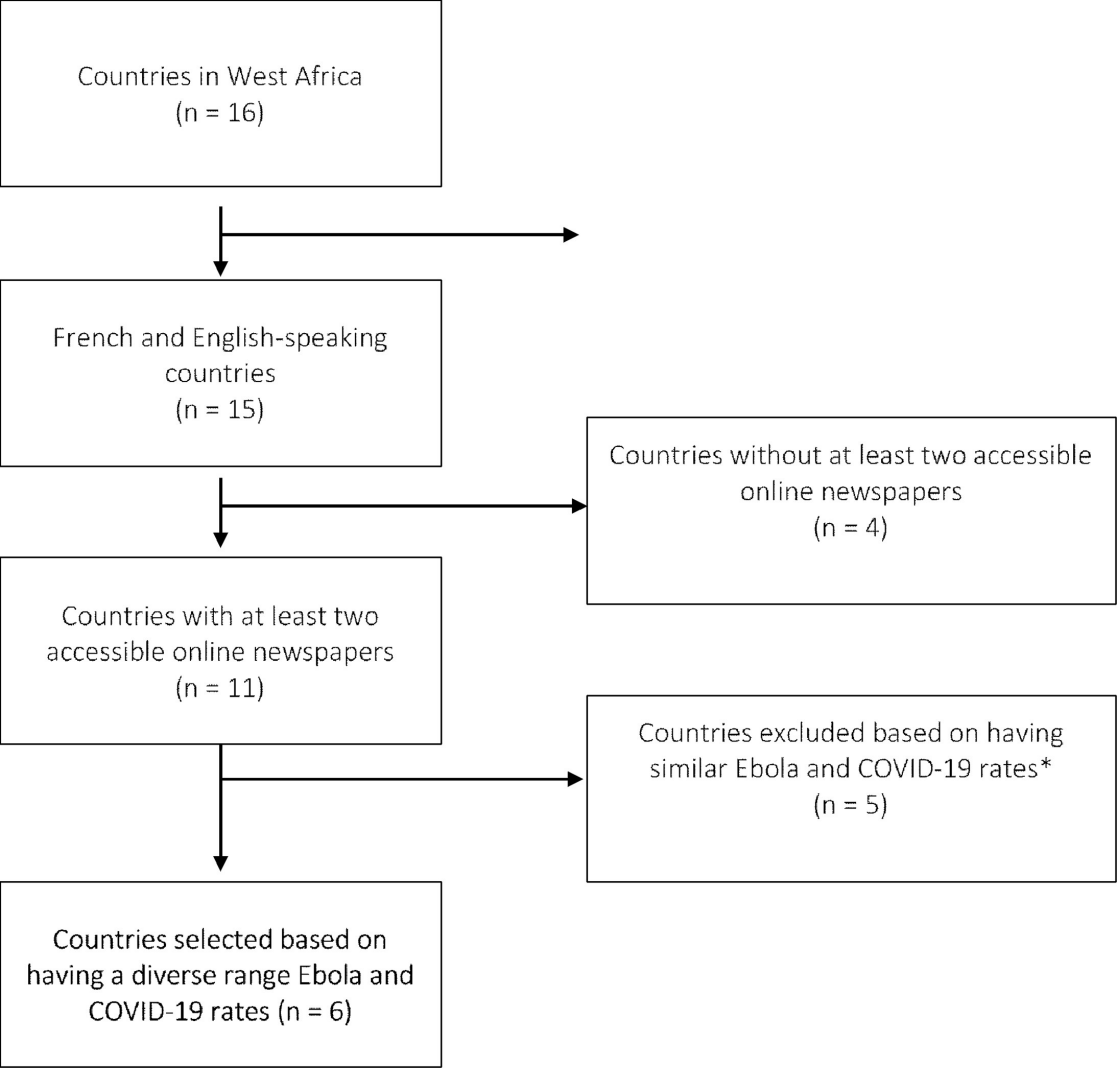
Country inclusion flow chart.

Countries were selected to reflect a diversity of experiences with Ebola and coronavirus, as well as accessibility of online news sources for the media (Fig 1). First, all 16 countries in West Africa were ranked by coronavirus incidence and case-fatality rates as of June 2nd, 2020 (S1, S2 Tables in S1 File) used as a proxy for diversity of COVID-19 experience. Second, the number of Ebola cases and deaths reported during the West Africa Ebola epidemic in each country was used to categorize countries by extent of experience with Ebola: no Ebola, moderate Ebola, and high Ebola. Countries with at least two available online news sources in either English or French were included in sampling frame. Maximum variation sampling was then employed to select six countries with the greatest range in levels of experience with coronavirus and Ebola [23]. The countries selected were Ghana, Guinea, Liberia, Niger, Nigeria, and Sierra Leone.

### Search strategy, data collection, and data abstraction

**Fig 2:**
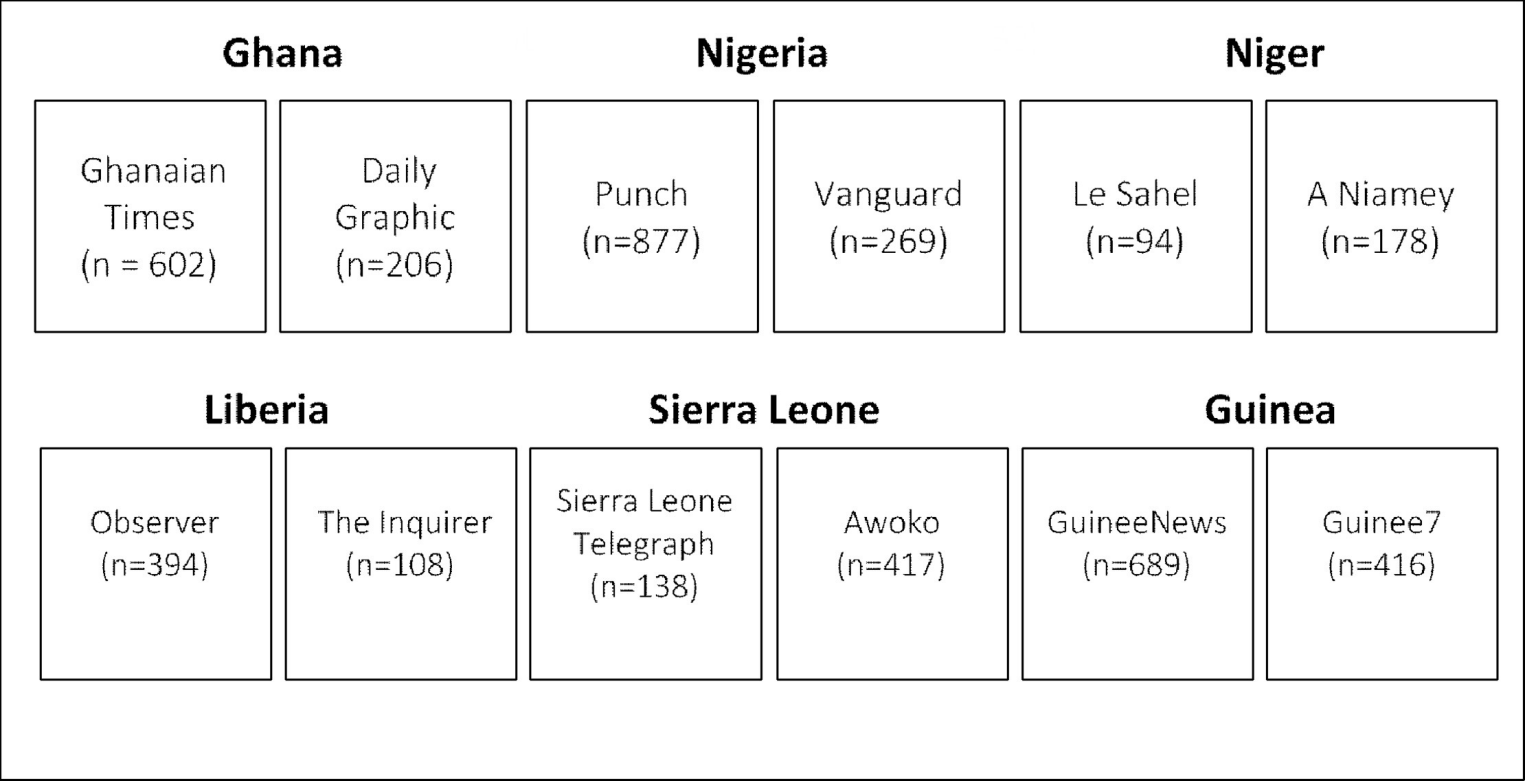
News article inclusion chart.

For each country, two leading news sources were selected based on circulation numbers (Fig 2). Circulation numbers were used to determine the most popular and most read online news sources within each country. For logistical reasons, only online newspapers were included. The list of news sources was a composite of both publicly and privately owned newspapers: *Ghanaian Times* (public, circulation: 80,000) [24] and *Daily Graphic* for Ghana (public, circulation: 100,000) [25]; *Daily Observer* (private, circulation: not found) [26] and *The Inquirer* for Liberia (private, circulation: not found) [27]; *Punch* (private, circulation: 80,000) [28] and *Vanguard* (private, circulation:120,000) [29] for Nigeria; *Sierra Leone Telegraph* (ownership not found, circulation: not found) [30] and *Awoko* (private, circulation: not found) [31] for Sierra Leone; *Le Sahel* (public, circulation: not found) [32] and *A Niamey* (private, circulation: not found) [33] for Niger; and *GuineeNews (*private, circulation: 245,000) [34] and *Guinee7* (ownership not found, circulation: not found) [35] for Guinea. Newspapers whose circulation numbers were not found were listed as the top read newspapers in country [36]. West Africa has high overall mobile phone ownership, with 86% of the population having a SIM connection, denoting internet access [37]. All news articles, regardless of classification, published from January 1 to June 30, 2020 and containing any of the search terms: COVID-19; COVID; Coronavirus; Coronavirus-19; nCov-19; Corona; SARS-CoV-2; Coronavirus-2019; and pandemic, were identified and downloaded directly from the news sites, and imported into Dedoose Version 8.0.35 [38]. Prior to importing to Dedoose, articles in French were translated into English using the Adobe translate plug-in, then the accuracy of the translation was verified by French-speaking study team members.

### Ethical considerations

Ethical approval was not required for this study. All data was obtained from news sources, information freely available in the public domain.

### Data processing and analysis

A mix of deductive and inductive coding was performed. Deductive codes were drawn from the World Health Organization (WHO)’s frameworks, WHO’s Everybody’s Business: Strengthening Health Systems to Improve Health Outcomes and Managing Epidemics [39, 40]. The documents provide a blueprint to evaluate the effects of the pandemic on core components of health systems and provide insight into standard approaches countries may use to effectively respond to the pandemic. To identify any remaining codes that were not included in the WHO frameworks, a random selection of 40 articles from all countries covering the period January through June was coded inductively.

Each team member was required to complete two evaluations of coding 40 sample news articles to assess intercoder agreement (kappa score) [41]. Team members were required to have a kappa score of 0.80 or above before starting to code. Further disagreements between the coders were resolved through discussion. The final codebook included codes focused on the existing effects of COVID-19 on vulnerable populations, which were defined inductively during the coding process as groups who were identified by the articles as at risk of experiencing more negative effects of COVID-19. Codes also focused on solutions that West African governments, organizations, and populations used to manage the effects of COVID-19. Only solutions that were implemented at the time the articles were written were included.

A total of 4,388 news articles were coded for mention of the existing effects of COVID-19 on the vulnerable populations including mothers and children, prisoners, older adults, and persons with disabilities, informal sector workers and poor populations and solutions used to mitigate these effects (S3 Table in S1 File). Excerpts from coded articles were categorized into themes by reported effects on vulnerable population and types of solutions implemented. For the purposes of this manuscript, the excerpts selected for inclusion were chosen by our analysis team as the most representative of each theme.

## Results

The coded news articles produced a total of 285 excerpts (S4, S5 Tables in S1 File).

### Effects of COVID-19 on vulnerable populations in West Africa

#### Effects of COVID-19 on mothers and children

Mothers and children were by far the most commonly discussed vulnerable group during the COVID-19 pandemic, with over 129 excerpts discussing them specifically. The main challenges faced by mothers and children were: 1) disruptions in education; 2) reduction in care seeking behavior and access to health care; 3) sexual abuse and other forms of domestic violence; and 4) food and economic insecurity.

*Disruptions in education*. From the beginning of the pandemic, school closings were reported across West Africa, potentially interrupting cognitive and psychological development (GU-C1089F). News outlets in Nigeria and Liberia frequently reported fears that the pandemic would worsen circumstances for the most at-risk students, particularly children with special needs and disabilities, who were more susceptible to drop out (LI-C0178E) (NA-C0497E).

“Many students are ‘at-risk’ of dropping out of school including students of low-income households, overage students, children with disabilities and those affected by one or a combination of factors such as from poor teacher attendance, overcrowding, sexual violence or the long commute to schools.” (LI-C0178E)

*Reduction in care seeking and access to health care*. In most countries, except Niger, articles documented that the pandemic reduced the level of preventative health care seeking behaviors for women and children. Liberia, Sierra Leone, Guinea, and Nigeria, countries with moderate to high previous Ebola experience, reported the most concern with access to health care for mothers and children. In Ghana and Sierra Leone, newspapers reported that some pregnant women refused to attend antenatal care and feared giving birth at health facilities due to concerns of contracting the virus (SL-C0459E). *Sierra Leone Telegraph* wrote:

“In Port Loko and the Western Area Urban, pregnant and lactating mothers abandoned hospitals prior to the lockdown for fear that they could be injected (infected) with the virus. This resulted in more health risks, as these women and their innocent children suffer in silence” (SL-C0459E).

In Guinea, mothers were hesitant to bring their children to health facilities to receive vaccinations for preventable diseases (GU-C0197F). Moreover, some vaccination programs halted in Guinea and Nigeria and in certain regions, health facilities were reported to have reduced the quota for the number of children to be vaccinated per day (GU-C0877F, NA-C0241E).

The pandemic additionally inhibited access to family planning products or services, as noted in Sierra Leone and Liberia. *The Inquirer* newspaper of Liberia reported that in one survey, 96% of women encountered challenges traveling to health facilities to receive family planning products because of the lockdown and fear of visiting health centers during the pandemic (LI-C0012E). Along with creating individual barriers to family planning, newspapers indicated that the pandemic triggered delays in production and shipment of family planning products to West African countries (LI-C0012E).

*Food and economic insecurity*. With the exception of Guinea and Nigeria, news articles in most countries reported that the COVID-19 pandemic exacerbated food and economic insecurity, particularly for children. With the closing of schools during the pandemic, many children were left to fend for themselves or obligated by their family to contribute to the household finances. According to some news sources, many children of low socio-economic backgrounds turned to the streets to sell items or to beg for money and food (SL-C0214E). In Liberia, children were seen on the street selling masks for an income without wearing any masks for protection, increasing their risk for contracting coronavirus (LI-C0085E).

Food and economic insecurity also hindered in-home learning for children in Sierra Leone as children spent more of their time selling on the streets, affecting their ability to attend implemented radio learning programs.

“Many children are not following the radio teaching programs initiated by the Teaching Service Commission (TSC)” Nicole noted. “I come to sell cold water every day from 8:00 a.m. and return home around 7:00 p.m. It is during this coronavirus pandemic that I started selling cold water,” a young boy said at Sani Abacha.” (SL-C0214E)

*Sexual abuse and other forms of domestic violence*. All countries reported serious concerns about the effects of COVID-19 on domestic violence and sexual abuse for women and children. The economic and psychosocial effects of COVID-19 and the additional burden of stay-at-home orders escalated tension in families and placed women and children at risk for abuse. In Liberia, where rates of gender-based violence were reported as high before the pandemic, the rise in violence cases was said to be alarming (LI-C0008E).

“Most of the women and girls no longer have anywhere to hide because most of the violence happens at home; a place which should be a safe space for any individual. It is frightening to imagine, that for many women and girls, homes are not being recognized as a ‘safe space’ but in captivity and violence with their abusers” (LI-C0008E).

As a result of school closures, many children transitioned to in-home learning, which for certain children increased their exposure to abuse that may have been occurring at home. In Nigeria, for example, newspapers documented serious concerns surrounding child safety and noted the disturbing rise in rape cases of children younger than 12 years old, including a case of a 4-year old child raped by a 70-year-old man (NA-C0029E).

#### Effects of COVID-19 on prisoners and prison staff

Prisoners and prison staff was another group discussed among the news articles. News articles in Liberia, Guinea, and Sierra Leone, all countries with previous Ebola experience, reported on the effect of COVID-19 on prisons. In Guinea, there were 58 coronavirus cases and 3 deaths in prisons by May (GU-C0440F, GU-C0448F, GU-C0450F), while there were 30 cases in the prisons in Sierra Leone by June (SL-C0471E). Guinea news sources had the most excerpts concerning prisoners and prison staff. The major negative effects reported were: 1) Overcrowded prisons and panic concerning COVID-19 and 2) lack of food and water.

*Overcrowded prisons and panic concerning COVID-19*. In news articles from Guinea, the word “psychosis” was used to capture the fear, confusion, and disconnect from reality that prisoners felt because of COVID-19. The term “psychotic” was also used to describe the environment in prisons following reports of the first coronavirus case in a prison (GU-C0106F). Detainees and staff feared the rapid spread of the virus in their prison because of having seen community members die from COVID-19 (GU-C0427F), the severely cramped living conditions (SL-C0235E), and the lack of sufficient information on the virus (GU-C0427F). Detainees and staff were even afraid of deaths that had no connection with coronavirus, leading to riots in some cases (SL-C0235E, GU-C0440F).

Newspaper sources indicated that most of the prisons in West Africa, especially in Liberia and Guinea, were already overcrowded and contained four to five times their detainee capacity, violating basic human rights (LI-C0604E, GU-C0103F). For example, in Liberia:

“The Monrovia Central Prison (MCP) that was originally built for 374 detainees, now holds 1,262 inmates” (LI-C0604E).

News sources also documented that the pandemic contributed to abuse of political power and inflated prison populations, specifically in Guinea. For example, the Guinean Government stepped up politically motivated arbitrary arrests (GU-C0460F), including executive members of the National Front for the Defense of the Constitution (FNDC). *GuineeNews* stated that the arrests were contrary to the WHO and the United Nations recommendations to the government to reduce the prison population due to this pandemic (GU-C0440F). This abuse of power in Guinea also drew the attention of Amnesty International (GU-C0450F).

*Lack of food and water*. In Sierra Leone and to a lesser extent Guinea, inadequate aid and preventative measures to fight against COVID-19 worsened the vulnerability of prisoners and staff to coronavirus. Due to overcrowding and water scarcity, prisoners and wardens were not able to take baths regularly or frequently wash hands as recommended to prevent from contracting the virus (SL-C0471E). *Sierra Leone Telegraph* wrote:

“Even as Coronavirus is spreading inside the Freetown prisons among prisoners, the authorities in Sierra Leone have left the prisoners without adequate water supply for the past ten days” (SL-C0471E).

The water crisis was so extreme in some of the prisons that prisoners were urinating and defecating inside their cell toilets with the inability to flush, forcing them to sleep without fresh air (SL-C0471E). Moreover, as a result of the prohibition on visits due to COVID-19, prisoners were concerned about getting enough food in an already precarious situation (SL-C0235E).

#### Effects of COVID-19 on older adults and adult persons with disabilities

Though less frequently mentioned, news organizations from Guinea, Liberia, Sierra Leone, and Niger reported on older adults and persons with disabilities as vulnerable groups. In the articles, these two groups were commonly associated because they were most likely to require extra physical assistance and care throughout the pandemic. The major effects discussed were poverty, stigma, and lack of access to assistance for both populations. There were too few excerpts on these groups to determine if reporting varied by Ebola experience.

*Poverty*, *stigma*, *and lack of access to assistance*. News articles reported on the poverty that affected both older adults and people living with a disability during this pandemic. *Daily Observer* of Liberia also reported that poverty was an additional vulnerability older adults faced during the pandemic as many of older adults and their family members were trapped at home due to the pandemic and unable to work (LI-C0165E). *Le Sahel* reported on the financial struggle of women and girls with disabilities:

“Women and girls with disabilities are at the lowest point of the poverty ladder, and COVID-19 is placing additional burdens on them, namely purchasing personal protective equipment and disinfectant products for themselves, people for which they are responsible, including personal assistants” (NR-C0063F).

COVID-19 mitigation measures such as social distancing and the use of masks worsened the situation for those with disabilities, as finding additional physical assistance or support from aides was difficult during the pandemic (NR-C0063F). However, these groups were often not consulted during policymaking and thus, were deprived of effectively benefitting from the special initiatives that governments took to fight COVID-19 (NR-C0063F).

#### Effects of COVID-19 on informal sector workers and poor populations

Informal sector workers including beggars, farmers, market sellers along with low-income families were recognized and reported on as a vulnerable group in Guinea, Ghana, Liberia, and Niger. Reporting on this population was variable across countries with and without Ebola experience. Effects discussed relating to this population were: 1) worsening food insecurity and 2) inability to sell products.

*Worsening food insecurity*. As a result of COVID-19, many low-income families experienced worsened food insecurity, according to the news sources in our sample. The *Daily Observer* in Liberia reported that most of their effects on food security were caused by local lockdowns and curfew orders:

“A total lock-down is not advisable because a large percentage of the nation’s population does not have the capacity to store up food for more than a day” (LI-C0631E).

*Daily Observer* also disclosed that some Liberian families reduced their daily meals from three per day to one per day to save food and about 60% of Liberians were beginning to starve (LI-C0785E). *Ghanaian Times* reported that food insecurity in Ghana was exacerbated because many food items in Ghana were imported from other countries (GH-C0515E). Guinea reported on the similar effect such closures had on beggars. *GuineeNews* reported:

“Among vulnerable groups, beggars—the majority of whom are disabled—have seen their situation deteriorate with the outbreak of COVID-19. This was mainly due to the closure of places of worship, especially mosques where they flocked in search of alms” (GU-C0472F).

*Inability to sell products*. News sources reported that COVID-19 affected the ability of persons to sell their produce and other products. Social distancing measures caused farmers to suffer from a lack of manpower to grow and sell their produce (LI-C0613E). Corn farmers in Guinea faced several issues in selling their produce including drops in the purchasing price of corn and hikes in transportation costs and market closures (GU-C0141F). Consequently, a large amount of produce perished causing tremendous losses in profits for farmers in Guinea (GU-C0511F). A member of a Guinean agricultural group described:

“Because of the disease, the local market was closed…You can’t keep ripe tomatoes, eggplants or okra, for example, once you pick them. Personally, I lost a lot during these weeks without a market.” (GU-C0511F)

In Liberia, in addition to farmers, vegetable sellers, whose customers were often from other areas, experienced a decrease in overall sales due to border closures (LI-C0613E).

### Solutions to manage the effects of COVID-19 on vulnerable populations

In 110 excerpts, solutions were described to manage the effects of COVID-19 on vulnerable populations, including local and foreign donations, technical assistance, technological innovation and study materials, and release of detainees.

#### Local and foreign donations

Donations from both local and international entities were distributed to relieve the negative effects of COVID-19 in West African countries, including money, food items, educational materials, medical products, and sanitation and personal protective equipment. Most donations were directed towards mothers and children and poor populations to reduce the potential food and economic insecurity brought on by the pandemic (SL-C0174E, LI-C0611E, GU-C0472F, GU-C0488F, GH-C0028E). Mothers and young women also received donations of family planning products and sanitary pads, and young children received various donations made to support continuous education, personal protective equipment, and educational materials for in-home learning. A few donations including sanitary kits for handwashing and food products were distributed to prisoners and prison staff and people with disabilities (NR-C0258F) (LI-C0034E, LI-C0004E). Older adults and people living with disabilities in Ghana received some of these donations in addition to masks and personal protective equipment (GH-C0476E).

However, according to *Le Sahel* of Niger, the COVID-19 emergency response team rarely directed the distribution of food and other personal protective equipment toward older adults and persons with disabilities (NR-C0063F).

#### Technical assistance to improve programming

Technical support, defined as guidance provided to governments by international and regional organizations, was provided to the governments of West Africa to alleviate food insecurity and economic vulnerability of mothers and children. For instance, in Nigeria, the World Food Programme offered the government strategic support to enhance their social protection systems, aiming to scale up the systems in communities with most financially unstable households during the pandemic (NA-C0074E). In Niger, technical assistance from the United Nations Population Fund (UNFPA) and the West African Health Organization (WAHO) was aimed at ensuring girls returned to school and the continuity of access to maternal and child health and family planning services (NR-C0004F).

#### Technological innovations and information for children

Innovations and technology were also used to lessen certain effects, such as via the rapid development of alternative approaches to learning for children throughout the pandemic. In Liberia, Nigeria, and Sierra Leone, international and local organizations supported programs promoting online schooling and radio programs (LI-C0178E, LI-C0610E, NA-C1144E, SL-C0517E). The government of Sierra Leone created an education emergency response for the students with special needs, providing preloaded devices and printed educational packages for these students as well as trained teachers to deliver lessons through digital platforms (SL-C0517E). An additional challenge cited was the limited capacity to transition to online learning because of limited technological infrastructure, inadequate faculty preparedness, and lack of access to reliable internet connectivity for students at home (GH-C0664E, LI-C0178E).

#### Maintaining access to coronavirus related prevention and health services

Measures were taken to maintain the continuity of health services during the pandemic. Certain organizations in Nigeria and Sierra Leone worked with people living with disabilities to ensure equity in access to the health care facility and other emergency services. Such organizations also promoted hand washing and social distancing among mentally challenged people living in slums and offering preventative materials for their protection (NR-C0071F, SL-C0205E). To protect the incarcerated population, the Guinean government implemented several preventative measures, including screening prisoners and staff and constructing temporary treatment and isolation centers (GU-C0440F, GU-C0698F). Sierra Leone assigned medical specialists to the prison population (SL-C0471E).

#### Release of detainees and protections for prisoners

To mitigate the spread of the virus, several news sources indicated that measures were taken to decongest prisons. Newspapers from four countries reported remission of sentences and release of prisoners (GU-C0278F, GU-C0440F). In Niger, over 1,500 convicts were released to allow the decongestion of prisons (NR-C0258F).

“Issoufou Mahamadou has decided to grant graceful remissions of the sentence to certain convicts whose remaining sentence is less than nine (9) months and people over seventy (70) years old. These individual discounts are also granted to vulnerable people such as women, minors, and people with serious or chronic illnesses” (NR-C0017F).

Despite calls to release the vulnerable and pre-trial detainees and take measures immediately to protect prisoners, responses were often taken too late to effectively protect prisoners and staff (LI-C0604E, SL-C0471E, SL-C0130E).

The Guinean government implemented several preventative measures to mitigate the effect of COVID-19 in prisons, including screening of 1,448 prisoners and their staff and the construction of treatment and temporary isolation centers inside the prison (GU-C0440F, GU-C0698F). Sierra Leone also made efforts to control the coronavirus cases in prisons by providing medical specialists to the prison population (SL-C0471E).

## Discussion

The findings of this media analysis emphasize the effects of the COVID-19 pandemic on the vulnerable groups including mothers and children, prisoners, older adults, persons with disabilities, and the poor in West Africa. Vulnerable populations in West Africa were reported to have experienced a range of effects including economic disruptions, human rights abuses, health care inaccessibility, and educational challenges throughout the pandemic, varying by group. Food insecurity and poverty were cross-cutting effects of the pandemic on these vulnerable populations. Mothers and children were the most mentioned vulnerable population throughout the excerpts. Although prisoners, older adults, people with disabilities, informal sector workers and poor populations were mentioned less frequently in news articles, excerpts on these populations were revealing about the insufficiency of measures taken to protect them. Overall, newspapers in countries with no past experience with Ebola contained fewer discussions of the effects on vulnerable populations. Of note, any mentions of the effect of COVID-19 on prisoners were only found in countries that had experienced the Ebola epidemic.

Our finding that COVID-19 disrupted the economic conditions of vulnerable populations is consistent with other studies calling attention to COVID-19’s effect on poverty in Sub-Saharan Africa and citing curfews and lockdowns as main contributing factors [42, 43]. In our media analysis, West African farmers were reportedly unable to farm and sell products because of COVID-19 restrictions, which heightened food insecurity and reduced income among the poorest populations. Women and children were reported to have also faced both worsened food insecurity and heightened risk for COVID-19 due to this insecurity. The economic effect of COVID-19 on vulnerable populations parallels the effects observed during the West Africa Ebola epidemic of 2014–2015, which also exacerbated food insecurity and loss of income [44, 45]. For instance, in Liberia, though agricultural households remained in operation, harvests were smaller than previous years and Liberian women, often traders or market sellers, experienced the worst rates of unemployment [44]. Combined with emerging evidence from the current pandemic, these findings demonstrate that health crises can perpetuate socio-economic vulnerabilities, and poverty-alleviating initiatives should be part of pandemic response.

Human rights abuses and unsafe conditions were reported for imprisoned people across most study countries. Notably, since the prison population is often neglected in the literature [46, 47], reports about prisoners were mostly made in the Guinea, Sierra Leone and Liberian newspapers, all countries that were also affected by the Ebola epidemic. Countries with previous experience of Ebola, and particularly Guinea, appear to have demonstrated an increased level of pandemic response in terms of protecting prisoners. Regardless of experience with Ebola, countries in our study appeared to have insufficient capacity to keep prisoners safe and manage the psychosocial effects of COVID-19, resulting in violence in prisons and a rise in coronavirus cases. Similar findings have been reported in other countries, such as Brazil, where reports of rebellion in prisons were linked to inadequate pandemic response, with prison occupancy being over 3 times its total capacity [48, 49]. In West Africa, our findings show that the human rights violations and prison riots were fueled by overcrowded prisons, unsanitary living conditions, and lack of COVID-19 prevention measures. Human rights violations were also reported to have been exacerbated by politically-motivated arbitrary arrests, such as in Guinea, and for arrests as a result of breaking curfew and lockdown orders. Relatedly, restricting visits to prisons is a common approach which countries globally are using to prevent COVID-19 in prisons [50, 51]. However, our analysis suggests that in resource-limited settings, this approach limits access to food and needed supplies from families, and in West Africa, it exposes prisoners to the full brunt of the human right abuses that occur in prisons.

Domestic violence against women, young girls, and children was another human rights concern that was observed throughout the pandemic in all study countries. These results are consistent with the existing literature, suggesting lessons learned from the rise in domestic violence during the Ebola crisis were not fully incorporated into the COVID-19 responses of these countries [52]. Our analysis suggests that few efforts to protect women and children were made in preparation for the lockdown during the COVID-19 pandemic. Government support of women and children, including protection against domestic violence as a specific measure in contingency plans against COVID-19, is required to ensure their access to legal support and police protection [53].

The challenge of healthcare inaccessibility was reported to have mostly affected mothers, children and persons with disabilities. Reduction in care-seeking behaviors for mothers and children were most reported in Sierra Leone and Guinea, countries with previous Ebola experience. In fact, the reported effects were similar to what was observed during the Ebola epidemic. During the Ebola outbreak of 2015, some studies indicated that maternal care and vaccinations for children drastically declined in Sierra Leone, Liberia, and Guinea [54–56]. In Sierra Leone, between May and September of 2014, the number of women attending health facilities for deliveries declined by 27% due to Ebola concerns [57]. It is possible that populations’ recollection of the fear of contracting Ebola at health facilities could have influenced care seeking practices during the COVID-19 pandemic, a potential explanation for the more frequent mentions of reduced care seeking during COVID-19 in countries with Ebola experience.

This analysis provides additional evidence that the closure of schools and transition to remote online learning during the COVID-19 pandemic may have stunted the educational development of children, especially those with disabilities [58, 59]. Our media analysis highlights the innovations that certain West African countries adopted to mitigate the effect of the pandemic on childhood education. While news articles reported on various measures taken to develop in-home learning technologies across all countries, Nigeria, Sierra Leone, and Liberian were the only countries to recognize the disproportionate effect of disrupted education on children with learning disabilities in the included news sources. Consequently, there was an overall gap of information on how these in-home learning technologies were adapted to children with disabilities throughout the news articles.

For solutions, though some donations have been earmarked for certain populations, the imbalance in donations reported by these news articles shows that West African countries may benefit from a more strategized allocation of donations. Some recommendations include encouraging local donations to target vulnerable populations who are the most overlooked by donor organizations, such as prisoners, older adults, and individuals with disabilities. Based on solutions reported, we recommend that these countries diversify types of aid rather than rely primarily on donations, such as request technical assistance, increase distribution of health information, and leverage existing technological opportunities for people living with disabilities.

This media analysis offers a broad understanding of the effects of COVID-19 on vulnerable populations in West Africa as reported by the news media, with the inclusion of six English- and French-speaking countries allowing for comparison between those with and without previous experience of Ebola. However, this study has some limitations. First, the effects of COVID-19 identified through a media analysis may not reflect the true individual and community level effects of the pandemic. Second, the selection of only the two most read online news sources per country without considering their ideologies and reporting practices may have influenced the outcomes of this study, resulting in selection bias. Third, not all vulnerable populations in West Africa were well covered in news articles, such as migrant workers, refugees, and sexual minorities. Fourth, only online news sources that were written in English or French were used, potentially excluding some relevant sources. Nevertheless, the news sources selected had the most online coverage throughout their respective countries. Lastly, this study only covered news articles published between January 1st and June 30th, 2020. Given the evolving nature of the pandemic, it is likely our findings are only representative of the initial effects and immediate responses to the pandemic.

## Conclusions

This study highlighted several commonalities among West African countries regarding the populations that were particularly vulnerable to the social effects of this pandemic, including a focus on women and children across all countries, with lesser attention to older adults, poor and persons with disabilities. Imprisoned people were granted greater, yet still insufficient, attention in countries with previous experience of Ebola. Given this knowledge, West African countries should consider and focus more strategic efforts on vulnerable populations to overcome their fight against the COVID-19 pandemic and to achieve the Sustainable Development Goals (SDG) for 2030. Further in-depth research is needed to provide a deeper understanding of the contextual factors behind the identified effects and to assess more long-term effects of the pandemic in West Africa and other regions.

## Supporting information

S1 File (DOCX)

## References

[1] Coronavirus COVID-19 (2019-nCoV) [Internet]. [cited 2021 Feb 9]. Available from: https://gisanddata.maps.arcgis.com/apps/opsdashboard/index.html#/bda7594740fd40299423467b48e9ecf6

[2] Africa CDC—COVID-19 Daily Updates [Internet]. Africa CDC. [cited 2021 Feb 9]. Available from: https://africacdc.org/covid-19/

[3] Atoui I, Boumediene A. COVID-19 casts shadow on future of West African countries [Internet]. Daily Sabah. 2020 [cited 2021 Feb 9]. Available from: https://www.dailysabah.com/opinion/op-ed/covid-19-casts-shadow-on-future-of-west-african-countries

[4] Soy A. Lack of Covid-19 testing undermines Africa’s “success”—BBC News [Internet]. [cited 2021 Feb 9]. Available from: https://www.bbc.com/news/world-africa-52801190

[5] Uyoga S, Adetifa IMO, Karanja HK, Nyagwange J, Tuju J, Wanjiku P, et al. Seroprevalence of anti–SARS-CoV-2 IgG antibodies in Kenyan blood donors. Science [Internet]. 2021 Jan 1 [cited 2021 Apr 22];371(6524):79–82. Available from: https://science.sciencemag.org/content/371/6524/79 doi: 10.1126/science.abe1916 33177105 PMC7877494

[6] Tso FY, Lidenge SJ, Peña PB, Clegg AA, Ngowi JR, Mwaiselage J, et al. High prevalence of pre-existing serological cross-reactivity against severe acute respiratory syndrome coronavirus-2 (SARS-CoV-2) in sub-Saharan Africa. International Journal of Infectious Diseases. 2021 Jan 1 [cited 2021 Feb 9];102:577–83. doi: 10.1016/j.ijid.2020.10.104 33176202 PMC7648883

[7] Malani A, Shah D, Kang G, Lobo GN, Shastri J, Mohanan M, et al. Seroprevalence of SARS-CoV-2 in slums versus non-slums in Mumbai, India. Lancet Glob Health [Internet]. 2021 Feb [cited 2021 Apr 22];9(2):e110–1. Available from: https://www.ncbi.nlm.nih.gov/pmc/articles/PMC7836622/ doi: 10.1016/S2214-109X(20)30467-8 33197394 PMC7836622

[8] Oyeniran OI, Chia T. Fighting the Coronavirus disease (Covid-19) pandemic: Employing lessons from the Ebola virus disease response. Ethics Med Public Health. 2020 Dec;15:100558. doi: 10.1016/j.jemep.2020.100558 32837995 PMC7332950

[9] Experts react: Understanding the conflict in Tigray [Internet]. Atlantic Council. 2020 [cited 2021 Feb 9]. Available from: https://www.atlanticcouncil.org/blogs/africasource/experts-react-understanding-the-conflict-in-tigray/

[10] Burke J. South Africa issues arrest warrant for ANC’s Ace Magashule [Internet]. the Guardian. 2020 [cited 2021 Feb 9]. Available from: http://www.theguardian.com/world/2020/nov/10/south-africa-issues-arrest-warrant-for-ancs-ace-magashule

[11] OECD. COVID-19 and Africa: Socio-economic implications and policy responses [Internet]. [cited 2021 Feb 9]. Available from: http://www.oecd.org/coronavirus/policy-responses/covid-19-and-africa-socio-economic-implications-and-policy-responses-96e1b282/

[12] Ozili PK. COVID-19 in Africa: Socio-economic Impact, Policy Response and Opportunities [Internet]. Rochester, NY: Social Science Research Network; 2020 [cited 2021 Feb 9]. Report No.: ID 3574767. Available from: https://papers.ssrn.com/abstract=3574767

[13] Nations United. Policy Brief: Impact of COVID-19 on Africa [Internet]. 2020 [cited 2021 Feb 9]. Available from: https://unsdg.un.org/sites/default/files/2020-05/Policy-brief-Impact-of-COVID-19-in-Africa.pdf

[14] Durizzo K, Asiedu E, Van der Merwe A, Van Niekerk A, Günther I. Managing the COVID-19 pandemic in poor urban neighborhoods: The case of Accra and Johannesburg. World Development [Internet]. 2021 Jan 1 [cited 2021 Apr 22];137:105175. Available from: https://www.sciencedirect.com/science/article/pii/S0305750X20303028 doi: 10.1016/j.worlddev.2020.105175 32904458 PMC7455159

[15] Fawole OI, Okedare OO, Reed E. Home was not a safe haven: women’s experiences of intimate partner violence during the COVID-19 lockdown in Nigeria. BMC Women’s Health [Internet]. 2021 Jan 20 [cited 2021 Apr 22];21(1):32. Available from: doi: 10.1186/s12905-021-01177-9 33472627 PMC7816140

[16] Colebunders R, Siewe Fodjo JN, Vanham G, Van den Bergh R. A call for strengthened evidence on targeted, non-pharmaceutical interventions against COVID-19 for the protection of vulnerable individuals in sub-Saharan Africa. Int J Infect Dis. 2020 Oct;99:482–4. doi: 10.1016/j.ijid.2020.08.060 32861825 PMC7451006

[17] Wallace LJ, Nouvet E, Bortolussi R, Arthur JA, Amporfu E, Arthur E, et al. COVID-19 in sub-Saharan Africa: impacts on vulnerable populations and sustaining home-grown solutions. Can J Public Health. 2020 Oct 1 [cited 2021 Feb 9];111(5):649–53. doi: 10.17269/s41997-020-00399-y 32845460 PMC7448701

[18] 2014–2016 Ebola Outbreak in West Africa | History | Ebola (Ebola Virus Disease) | CDC [Internet]. 2020 [cited 2021 Feb 9]. Available from: https://www.cdc.gov/vhf/ebola/history/2014-2016-outbreak/index.html

[19] Armitage R, Nellums LB. Considering inequalities in the school closure response to COVID-19. The Lancet Global Health [Internet]. 2020 May 1 [cited 2021 Apr 22];8(5):e644. Available from: https://www.thelancet.com/journals/langlo/article/PIIS2214-109X(20)30116-9/abstract doi: 10.1016/S2214-109X(20)30116-9 32222161 PMC7195275

[20] Coltart CEM, Lindsey B, Ghinai I, Johnson AM, Heymann DL. The Ebola outbreak, 2013–2016: old lessons for new epidemics. Philos Trans R Soc Lond B Biol Sci [Internet]. 2017 May 26 [cited 2021 Apr 22];372(1721). Available from: https://www.ncbi.nlm.nih.gov/pmc/articles/PMC5394636/ doi: 10.1098/rstb.2016.0297 28396469 PMC5394636

[21] Nyenswah T, Blackley DJ, Freeman T, Lindblade KA, Arzoaquoi SK, Mott JA, et al. Community Quarantine to Interrupt Ebola Virus Transmission—Mawah Village, Bong County, Liberia, August–October, 2014. MMWR Morb Mortal Wkly Rep [Internet]. 2015 Feb 27 [cited 2021 Apr 22];64(7):179–82. Available from: https://www.ncbi.nlm.nih.gov/pmc/articles/PMC5779591/ 25719679 PMC5779591

[22] Macnamara J. Media Content Analysis: Its Uses, Benefits and Best Practice Methodology. Asia-Pacific Public Relations Journal. 2005 Jan 1;6.

[23] Palinkas LA, Horwitz SM, Green CA, Wisdom JP, Duan N, Hoagwood K. Purposeful Sampling for Qualitative Data Collection and Analysis in Mixed Method Implementation Research. Adm Policy Ment Health. 2015 Sep 1 [cited 2021 Feb 9];42(5):533–44. doi: 10.1007/s10488-013-0528-y 24193818 PMC4012002

[24] Ghanaian Times [Internet]. [cited 2021 Feb 9]. Available from: https://www.ghanaiantimes.com.gh/

[25] Daily Graphic [Internet]. [cited 2021 Feb 9]. Available from: https://www.graphic.com.gh

[26] Daily Observer [Internet]. [cited 2021 Feb 9]. Available from: https://www.liberianobserver.com

[27] The Inquirer [Internet]. [cited 2021 Feb 9]. Available from: https://www.theinquirernewspaper.com

[28] Punch [Internet]. [cited 2021 Feb 9]. Available from: https://punchng.com

[29] Vanguard [Internet]. [cited 2021 Feb 9]. Available from: https://www.vanguardngr.com

[30] Sierra Leone Telegraph [Internet]. [cited 2021 Feb 9]. Available from: https://www.thesierraleonetelegraph.com

[31] Awoko [Internet]. [cited 2021 Feb 9]. Available from: http://www.awoko.org/

[32] Le Sahel [Internet]. [cited 2021 Feb 9]. Available from: http://www.lesahel.org

[33] A Niamey [Internet]. [cited 2021 Feb 9]. Available from: http://news.aniamey.com

[34] GuineeNews [Internet]. [cited 2021 Feb 9]. Available from: https://www.guineenews.org

[35] Guinee7 [Internet]. [cited 2021 Feb 9]. Available from: https://www.guinee7.com

[36] All You Can Read [Internet]. [cited 2021 Feb 9]. Available from: https://www.allyoucanread.com/guinea-newspapers/

[37] GSM Association. The Mobile Economy West Africa [Internet]. 2019 [cited 2021 Feb 9]. Available from: https://www.gsma.com/mobileeconomy/wp-content/uploads/2020/03/GSMA_MobileEconomy2020_West_Africa_ENG.pdf

[38] Dedoose Version 8.0.35, web application for managing, analyzing, and presenting qualitative and mixed method research data. 2018 [cited 2021 Feb 9]. Los Angeles, CA: SocioCultural Research Consultants, LLC Available from: www.dedoose.com.

[39] World Health Organization. Everybody’s Business: Strengthening Health Systems to Improve Health Outcomes [Internet]. 2007 [cited 2021 Feb 9]. Available from: https://www.who.int/healthsystems/strategy/everybodys_business.pdf

[40] World Health Organization. Managing Epidemics [Internet]. 2018 [cited 2021 Feb 9]. Available from: https://www.who.int/emergencies/diseases/managing-epidemics/en/

[41] McHugh ML. Interrater reliability: the kappa statistic. Biochem Med (Zagreb) [Internet]. 2012 Oct 15 [cited 2021 Feb 9];22(3):276–82. Available from: https://www.ncbi.nlm.nih.gov/pmc/articles/PMC3900052/ 23092060 PMC3900052

[42] Chirisa I, Mutambisi T, Chivenge M, Mabaso E, Matamanda AR, Ncube R. The urban penalty of COVID-19 lockdowns across the globe: manifestations and lessons for Anglophone sub-Saharan Africa. GeoJournal [Internet]. 2020 Aug 27 [cited 2021 Feb 9]. Available from: doi: 10.1007/s10708-020-10281-6 32868960 PMC7450483

[43] Zeufack AG, Calderon C; Kambou G, Djiofack CZ, Kubota M, Korman V et al. Africa’s Pulse: An Analysis of Issues Shaping Africa’s Economic Future [Internet]. World Bank 2020; 21 [cited 2021 Feb 9]. Available from: https://openknowledge.worldbank.org/handle/10986/33541

[44] The World Bank, Liberian Institute of Statistics, Gallup. The Socio-economic Impacts of Ebola in Liberia [Internet]. 2015 [cited 2021 Feb 9]. Available from: https://www.worldbank.org/content/dam/Worldbank/document/Poverty%20documents/Socio-Economic%20Impacts%20of%20Ebola%20in%20Liberia%2C%20April%2015%20(final).pdf

[45] The World Bank, Sierra Leone Statistics. The Socio-Economic Impacts of Ebola in Sierra Leone [Internet] 2015 [cited 2021 Feb 9]. Available from: http://documents1.worldbank.org/curated/en/873321467999676330/pdf/97392-WP-P151624-Box391466B-PUBLIC-Socio-Economic-Impacts-of-Ebola-in-Sierra-Leone-June-2015-final.pdf

[46] Shadmi E, Chen Y, Dourado I, Faran-Perach I, Furler J, Hangoma P, et al. Health equity and COVID-19: global perspectives. International Journal for Equity in Health. 2020 Jun 26 [cited 2021 Feb 9];19(1):104. doi: 10.1186/s12939-020-01218-z 32586388 PMC7316580

[47] Stewart A, Cossar R, Stoové M. The response to COVID-19 in prisons must consider the broader mental health impacts for people in prison. Australian & New Zealand Journal of Psychiatry. 2020 Dec;54(12):1227–8. doi: 10.1177/0004867420937806 32571078 PMC7312101

[48] Matos MA. New Coronavirus (SARS-CoV-2): advances to flatten the curve the prison population. Revista da Sociedade Brasileira de Medicina Tropical [Internet]. 2020 [cited 2021 Feb 9];53. Available from: http://www.scielo.br/scielo.php?script=sci_abstract&pid=S0037-86822020000100909&lng=en&nrm=iso&tlng=en doi: 10.1590/0037-8682-0219-2020 32491063 PMC7269526

[49] “We’re all on death row now”: Latin America’s prisons reel from Covid-19 [Internet]. the Guardian. 2020 [cited 2021 Feb 9]. Available from: http://www.theguardian.com/world/2020/may/16/latin-america-prisons-covid-19-riots

[50] Iglesias-Osores S. Transmission and prevention of SARS-CoV-2 (COVID-19) in prisons. Revista Española de Sanidad Penitenciaria. 2020 May;22(2):87. doi: 10.18176/resp.00015 32697279 PMC7537360

[51] Heard C. Assessing the global impact of the Covid-19 pandemic on prison populations. Victims & Offenders. 2020 Oct 20.

[52] John N, Casey SE, Carino G, McGovern T. Lessons never learned: crisis and gender‐based violence. Developing world bioethics. 2020 Jun;20(2):65–8. doi: 10.1111/dewb.12261 32267607 PMC7262171

[53] Moreira DN, da Costa MP. The impact of the Covid-19 pandemic in the precipitation of intimate partner violence. International journal of law and psychiatry. 2020 Jul 1;71:101606. doi: 10.1016/j.ijlp.2020.101606 32768122 PMC7318988

[54] Sun X, Samba TT, Yao J, Yin W, Xiao L, Liu F, Liu X, Zhou J, Kou Z, Fan H, Zhang H. Impact of the Ebola outbreak on routine immunization in western area, Sierra Leone-a field survey from an Ebola epidemic area. BMC Public Health. 2017 Dec;17(1):1–6. doi: 10.1186/s12889-016-3954-4 28446173 PMC5406892

[55] Shannon FQ, Horace-Kwemi E, Najjemba R, Owiti P, Edwards J, Shringarpure K, Bhat P, Kateh FN. Effects of the 2014 Ebola outbreak on antenatal care and delivery outcomes in Liberia: a nationwide analysis. Public health action. 2017 Jun 21;7(1):S88–93. doi: 10.5588/pha.16.0099 28744445 PMC5515570

[56] Delamou A, El Ayadi AM, Sidibe S, Delvaux T, Camara BS, Sandouno SD, Beavogui AH, Rutherford GW, Okumura J, Zhang WH, De Brouwere V. Effect of Ebola virus disease on maternal and child health services in Guinea: a retrospective observational cohort study. The Lancet Global Health. 2017 Apr 1;5(4):e448–57. doi: 10.1016/S2214-109X(17)30078-5 28237252 PMC6530984

[57] Quaglio G, Pizzol D, Bome D, Kebbie A, Bangura Z, Massaquoi V, Frasson C, Dalla Riva D, Putoto G. Maintaining maternal and child health services during the Ebola outbreak: experience from Pujehun, Sierra Leone. PLoS currents. 2016 Jun 2;8. doi: 10.1371/currents.outbreaks.d67aea257f572201f835772d7f188ba5 28503359 PMC5419839

[58] Global Business Coalition for Education. Ebola Emergency: Restoring Education, Creating Safe Schools, and Preventing a Long-Term Crisis [Internet]. 2014 [cited 2021 Feb 9]. Available from: https://gbc-education.org/wp-content/uploads/2018/09/EbolaandEducationReport122014.pdf

[59] United Nations. Policy Brief: A Disability-Inclusive Response to COVID-19 [Internet]. 2020 [cited 2021 Feb 9]. Available from: https://unsdg.un.org/sites/default/files/2020-05/Policy-Brief-A-Disability-Inclusive-Response-to-COVID-19.pdf

