# Supplementary material for: Reported health and social consequences of the COVID-19 pandemic on vulnerable populations and implemented solutions in six West African countries: A media content analysis
Source: PLoS One. 2021 Jun 16;16(6):e0252890. doi: 10.1371/journal.pone.0252890 (PMC8208543; doi:10.1371/journal.pone.0252890)
Supplement: S1 File — (DOCX) [file pone.0252890.s001.docx]

### S1 Table. Countries by Ebola Status and COVID-19 Incidence Rates, as of June 2, 2020.

| **NO EBOLA** | **MODERATE EBOLA** | **HIGH EBOLA** |
| --- | --- | --- |
| The Gambia [1.1]  Benin [1.8]  **Niger [4.4]**  Burkina Faso [4.3]  Togo [5.0]  Mauritania [6.6]  Côte d’Ivoire [11.0]  Cameroon [21.6]  **Ghana [27.9]**  Guinea-Bissau [67.0] | **Nigeria [5.4]**  Mali [6.6]  **Guinea** **[8.4]** | **Liberia [6.1]**  **Sierra Leone** **[10.7]** |

Country [COVID-19 cases per 100,000 population]

### S2 Table. Countries by Ebola Status and COVID-19 Case Fatality Rate, as of June 2, 2020.

| **NO EBOLA** | **MODERATE EBOLA** | **HIGH EBOLA** |
| --- | --- | --- |
| **Ghana** **[0.5%]**  Guinea-Bissau [0.6%]  Benin [1.4%]  Côte d’Ivoire [1.2%]  Cameroon [3.2%]  Togo [3.3%]  The Gambia [4.0%]  Mauritania [5.5%]  Burkina Faso [6.3%]  **Niger** **[6.8%]** | **Guinea [0.6%]**  **Nigeria** **[3.0%]**  Mali [6.2%] | **Sierra Leone [5.4%]**  **Liberia [9.1%]** |

Country [COVID-19 case fatality rate]

### S3 Table. List of News Articles Referenced.

| **ID** | **Publication Month** | **Newspaper Name** | **Title of News Article** | **Link to News Article** | **Country** | **Language** | **Ebola vs. Non-Ebola** |
| --- | --- | --- | --- | --- | --- | --- | --- |
| GU-C0103F | April | GuineeNews | Mort des détenus à la Maison centrale : « c’est prématuré et hasardeux de conclure à une  pandémie », soutient la Justice | <https://www.guineenews.org/mort-des-detenus-a-la-maison-centrale-cest-premature-et-hasardeux-de-conclure-a-une-pandemie-soutient-la-justice/> | Guinea | French | Ebola |
| GU-C0106F | April | GuineeNews | Urgent : deux cas de décès enregistrés ce mardi à la Maison centrale de Conakry | <https://www.guineenews.org/urgent-deux-cas-de-deces-enregistres-ce-mardi-a-la-maison-centrale-de-conakry/> | Guinea | French | Ebola |
| GU-C0120F | May | GuineeNews | Lutte contre le coronavirus : Une quarantaine de ministres de 59 pays plaide en faveur des couches vulnérables (déclaration) | <https://www.guineenews.org/lutte-contre-le-coronavirus-une-quarantaine-de-ministres-de-59-pays-plaide-en-faveur-des-couches-vulnerables-declaration/> | Guinea | French | Ebola |
| GU-C0141F | May | GuineeNews | Baisse du prix des céréales à Lola : le cri du cœur des producteurs locaux | <https://www.guineenews.org/baisse-du-prix-des-cereales-a-lola-le-cri-du-coeur-des-producteurs-locaux/> | Guinea | French | Ebola |
| GU-C0197F | June | GuineeNews | Coronaphobie et les hôpitaux : un champ de foire pour la pharmacopée ! | <https://www.guineenews.org/coronaphobie-et-les-hopitaux-un-champ-de-foire-pour-la-pharmacopee/> | Guinea | French | Ebola |
| GU-C0278F | April | GuineeNews | Covid-19 et décongestion des prisons : la démarche « désespérée » des avocats de  Toumba | <https://www.guineenews.org/covid-19-et-decongestion-des-prisons-la-demarche-desesperee-des-avocats-de-toumba/> | Guinea | French | Ebola |
| GU-C0380F | April | GuineeNews | Maison centrale de Conakry: un cas de décès lié au Covid-19 signalé (Justice) | <https://www.guineenews.org/maison-centrale-de-conakry-un-cas-de-deces-lie-au-covid-19-signale-justice/> | Guinea | French | Ebola |
| GU-C0427F | May | GuineeNews | Guinée – COVID-19 : 33 nouveaux cas enregistrés et 11 morts | <https://www.guineenews.org/guinee-covid-19-33-nouveaux-cas-enregistres-et-11-morts/> | Guinea | French | Ebola |
| GU-C0440F | May | GuineeNews | Monde carcéral : vers la libération de plusieurs détenus pour cause du COVID-19 (Ociel) | <https://www.guineenews.org/monde-carceral-vers-lacquittement-de-plusieurs-detenus-pour-cause-du-covid-19-officiel/> | Guinea | French | Ebola |
| GU-C0448F | May | GuineeNews | Covid-19/Maison centrale de Conakry : l’OGDH ne crache pas sur le centre de prise en charge | <https://www.guineenews.org/covid-19-maison-centrale-de-conakry-logdh-ne-crache-pas-sur-le-centre-de-prise-en-charge/> | Guinea | French | Ebola |
| GU-C0450F | May | GuineeNews | En Guinée, les autorités alimentent le cycle de violence malgré le Covid-19 (Amnesty) | <https://www.guineenews.org/en-guinee-les-autorites-alimentent-le-cycle-de-violence-malgre-le-covid-19-amnesty/> | Guinea | French | Ebola |
| GU-C0457F | May | GuineeNews | Covid-19 en Guinée : Alpha Condé allège le couvre-feu de 22 heures à 5 heures du matin à Conakry (discours) | <https://www.guineenews.org/covid-19-en-guinee-alpha-conde-allege-le-couvre-feu-de-22heures-a-5-heures-du-matin-a-conakry-discours/> | Guinea | French | Ebola |
| GU-C0460F | May | GuineeNews | Revue de presse : Covid-19, crise politique, émeutes de Coyah, Kamsar et Coyah, le rapport CESA | <https://www.guineenews.org/revue-de-presse-covid-19-crise-politique-emeutes-de-coyah-kamsar-et-coyah-le-rapport-cesa/> | Guinea | French | Ebola |
| GU-C0468F | May | GuineeNews | Après avoir perdu son premier enfant, malgré le COVID-19, Sitan décide cette fois d’éviter le pire | <https://www.guineenews.org/apres-avoir-perdu-son-premier-enfant-malgre-le-covid-19-sitan-decide-cette-fois-deviter-le-pire/> | Guinea | French | Ebola |
| GU-C0469F | May | GuineeNews | COVID-19, Maria et Binta retrouvent le sourire après la prise en charge de leurs enfants à l’INSE | <https://www.guineenews.org/covid-19-maria-et-binta-retrouvent-le-sourire-apres-la-prise-en-charge-de-leurs-enfants-a-linse/> | Guinea | French | Ebola |
| GU-C0472F | May | GuineeNews | Covid-19 en Guinée : dans l’univers des mendiants, les autres victimes de la pandémie | <https://www.guineenews.org/covid-19-en-guinee-dans-lunivers-des-mendiants-les-autres-victimes-de-la-pandemie/> | Guinea | French | Ebola |
| GU-C0488F | May | GuineeNews | Covid-19 à Kankan : SOS Villages d’Enfants offer d’importants lots de vivres à des familles démunies | <https://www.guineenews.org/covid-19-a-kankan-sos-villages-denfants-offre-dimportants-lots-de-vivres-a-des-familles-demunies/> | Guinea | French | Ebola |
| GU-C0490F | May | GuineeNews | Guinée : après Conakry, la prison de Kindia enregistre son premier cas de décès dû au Covid-19 | <https://www.guineenews.org/guinee-apres-conakry-la-prison-de-kindia-enregistre-son-premier-cas-de-deces-du-au-covid-19/> | Guinea | French | Ebola |
| GU-C0511F | June | GuineeNews | Agriculture : La Covid-19 freine l’écoulement des produits agricoles au grand dam du monde paysan | <https://www.guineenews.org/agriculture-la-covid-19-freine-lecoulement-des-produits-agricoles-au-grand-dam-du-monde-paysan/> | Guinea | French | Ebola |
| GU-C0519F | June | GuineeNews | Riposte au Covid-19 : SOS villages d’enfants Guinée au secours de ses partenaires de Labé | <https://www.guineenews.org/riposte-au-covid-19-sos-villages-denfants-guinee-au-secours-de-ses-partenaires-de-labe/> | Guinea | French | Ebola |
| GU-C0692F | May | Guinee7 | CTE du COVID-19 à la Maison centrale: Pour les défenseurs des droits de l’homme, c’est bien ; mais il faut... | <https://www.guinee7.com/cte-du-covid-19-a-la-maison-centrale-pour-les-defenseurs-des-droits-de-lhomme-cest-bien-mais-il-faut/> | Guinea | French | Ebola |
| GU-C0693F | May | Guinee7 | Guinée : Selon Amnesty International, les autorités alimentent le cycle de la répression dans le context du COVID-19 | <https://www.guinee7.com/guinee-selon-amnesty-international-les-autorites-alimentent-le-cycle-de-la-repression-dans-le-contexte-du-covid-19/> | Guinea | French | Ebola |
| GU-C0698F | May | Guinee7 | Covid-19 : Un centre de prise en charge construit à la prison centrale de Conakry | <https://www.guinee7.com/covid-19-un-centre-de-prise-en-charge-construit-a-la-prison-centrale-de-conakry/> | Guinea | French | Ebola |
| GU-C0834F | June | Guinee7 | COVID19 : 28 cas positifs découverts à la prison de Kindia (ociel) | <https://www.guinee7.com/covid19-28-cas-positifs-decouverts-a-la-prison-de-kindia-officiel/> | Guinea | French | Ebola |
| GU-C0877F | May | Guinee7 | Kindia/ « Nous ne sommes pas bien outillés contre le COVID19 » Alpha Ousmane Barry, chef du centre de santé de Manquepas | <https://www.guinee7.com/kindia-nous-ne-sommes-pas-bien-outilles-contre-le-covid19-alpha-ousmane-barry-chef-du-centre-de-sante-de-manquepas/> | Guinea | French | Ebola |
| GU-C1089F | June | GuineeNews | Guinée : la Journée des Villages d’Enfants SOS célébrée sur fond de plaidoyer en faveur des enfants en cette période de Covid-19 | <https://www.guineenews.org/guinee-la-journee-des-villages-denfants-sos-celebree-sur-fond-de-plaidoyer-en-faveur-des-enfants-en-cette-periode-de-covid-19/> | Guinea | French | Ebola |
| GH-C0028E | March | Ghanaian Times | COVID-19: Cheddar Donates to Disabled and Needy in Accra | <https://www.ghanaiantimes.com.gh/covid-19-cheddar-donates-to-disabled-and-needy-in-accra/> | Ghana | English | Non-Ebola |
| GH-C0102E | March | Ghanaian Times | Students urged to adhere to Covid-19 prevention measures | <https://www.ghanaiantimes.com.gh/students-urged-to-adhere-to-covid-19-prevention-measures/> | Ghana | English | Non-Ebola |
| GH-C0169E | April | Ghanaian Times | COVID-19: Use continuous assessment to grade BECE, WASSCE students - CRI suggests to govt | <https://www.ghanaiantimes.com.gh/covid-19-use-continuous-assessment-to-grade-bece-wassce-students-cri-suggests-to-govt/> | Ghana | English | Non-Ebola |
| GH-C0182E | April | Ghanaian Times | COVID-19: BEHIND THE NUMBERS | <https://www.ghanaiantimes.com.gh/covid-19-behind-the-numbers/> | Ghana | English | Non-Ebola |
| GH-C0283E | May | Ghanaian Times | Guard against gender violence amid COVID-19- Prof. Kwankye | <https://www.ghanaiantimes.com.gh/guard-against-gender-violence-amid-covid-19-prof-kwankye/> | Ghana | English | Non-Ebola |
| GH-C0335E | May | Ghanaian Times | COVID-19: Bonabear Foundation | <https://www.ghanaiantimes.com.gh/covid-19-bonabear-foundation/> | Ghana | English | Non-Ebola |
| GH-C0385E | May | Ghanaian Times | More than 127m children out of school in Africa due to COVID-19 | <https://www.ghanaiantimes.com.gh/more-than-127m-children-out-of-school-in-africa-due-to-covid-19/> | Ghana | English | Non-Ebola |
| GH-C0432E | May | Ghanaian Times | UNICEF donates sanitary items, PPE to needy institutions | <https://www.ghanaiantimes.com.gh/unicef-donates-sanitary-items-ppe-to-needy-institutions/> | Ghana | English | Non-Ebola |
| GH-C0476E | June | Ghanaian Times | UCSOND provides PPEs to vulnerable, institutions in Nzemaland | <https://www.ghanaiantimes.com.gh/ucsond-provides-ppes-to-vulnerable-institutions-in-nzemaland/> | Ghana | English | Non-Ebola |
| GH-C0515E | June | Ghanaian Times | COVID-19: A war against hunger and malnutrition | <https://www.ghanaiantimes.com.gh/covid-19-a-war-against-hunger-and-malnutrition/> | Ghana | English | Non-Ebola |
| GH-C0581E | May | Graphic | Ghanaian entrepreneur wins UK grant to fight coronavirus across Africa - | <https://www.graphic.com.gh/business/business-news/ghanaian-entrepreneur-wins-uk-grant-to-fight-coronavirus-across-africa.html> | Ghana | English | Non-Ebola |
| GH-C0614E | June | Graphic | Matilda Agamu, the first Upper East COVID-19 recovered patient delivers baby safely | <https://www.graphic.com.gh/news/general-news/matilda-agamu-the-first-upper-east-covid-19-recovered-patient-delivers-baby-safely.html> | Ghana | English | Non-Ebola |
| GH-C0656E | June | Ghanaian Times | GES: Parents must assist children to observe COVID-19 protocols | <https://www.ghanaiantimes.com.gh/ges-parents-must-assist-children-to-observe-covid-19-protocols/> | Ghana | English | Non-Ebola |
| GH-C0664E | June | Ghanaian Times | Impact of COVID-19 on Higher Education in Africa: The Transition to Online Teaching and Learning | <https://www.ghanaiantimes.com.gh/impact-of-covid-19-on-higher-education-in-africa-the-transition-to-online-teaching-and-learning/> | Ghana | English | Non-Ebola |
| GH-C0788E | June | Graphic | Kids exposed to Covid-19 through hawking | <https://www.graphic.com.gh/junior-graphic/junior-news/kids-exposed-to-covid-19-through-hawking.html> | Ghana | English | Non-Ebola |
| LI-C0004E | June | The Inquirer | Jeety Gives To Central Prison | <https://www.theinquirernewspaper.com/amp/jeety-gives-to-central-prison/> | Liberia | English | Ebola |
| LI-C0008E | June | The Inquirer | Press Release- Spotlight Initiative Supports Media Training On Gender Sensitive Reporting On COVID-19 And SGBV In Liberia | <https://www.theinquirernewspaper.com/press-release-spotlight-initiative-supports-media-training-on-gender-sensitive-reporting-on-covid-19-and-sgbv-in-liberia/> | Liberia | English | Ebola |
| LI-C0012E | May | The Inquirer | DKT Liberia Innovates Its Actions To Mitigate Covid-19 Impact On Access To Family Planning | <https://www.theinquirernewspaper.com/dkt-liberia-innovates-its-actions-to-mitigate-covid-19-impact-on-access-to-family-planning/> | Liberia | English | Ebola |
| LI-C0013E | May | The Inquirer | Education Finalizing Plan For Reopening Of Schools | <https://www.theinquirernewspaper.com/education-finalizing-plan-for-reopening-of-schools/> | Liberia | English | Ebola |
| LI-C0016E | May | The Inquirer | Where Is The Voice For The Liberian Students??? | <https://www.theinquirernewspaper.com/where-is-the-voice-for-the-liberian-students/> | Liberia | English | Ebola |
| LI-C0027E | May | The Inquirer | ORANGE LIBERIA MAKES ADDITIONAL COVID-19 RELIEF DONATIONS | <https://www.theinquirernewspaper.com/orange-liberia-makes-additional-covid-19-relief-donations/> | Liberia | English | Ebola |
| LI-C0034E | May | The Inquirer | Bropleh Donates To Bassa | <https://www.theinquirernewspaper.com/bropleh-donates-to-bassa/> | Liberia | English | Ebola |
| LI-C0081E | June | The Inquirer | Leo Simpson Proposes Appropriate Learning Activities …Writes President Weah | <https://www.theinquirernewspaper.com/leo-simpson-proposes-appropriate-learning-activities-writes-president-weah/> | Liberia | English | Ebola |
| LI-C0085E | June | The Inquirer | Liberia Joins Other Nations To Celebrate World Day Against Child Labor | <https://www.theinquirernewspaper.com/liberia-joins-other-nations-to-celebrate-world-day-against-child-labor/> | Liberia | English | Ebola |
| LI-C0095E | June | The Inquirer | Education On Plans To Reopen … says schools shall not charge new fees other than the balances/arrears | <https://www.theinquirernewspaper.com/education-on-plans-to-reopen-says-schools-shall-not-charge-new-fees-other-than-the-balances-arrears/> | Liberia | English | Ebola |
| LI-C0103E | June | The Inquirer | Ministry Of Education Outlines Decision On Retention And Promotion Statements As Well As Plans To Reopen Schools Ahead Of WASSCE | <https://www.theinquirernewspaper.com/ministry-of-education-outlines-decision-on-retention-and-promotion-statements-as-well-as-plans-to-reopen-schools-ahead-of-wassce/> | Liberia | English | Ebola |
| LI-C0117E | June | Daily Observer | Jeety’s COVID-19 Feeding Initiative Reaches 95K Mark | <https://www.liberianobserver.com/news/jeetys-covid-19-feeding-initiative-reaches-95k-mark/> | Liberia | English | Ebola |
| LI-C0122E | June | Daily Observer | The Country Is Pulling Together to Tackle COVID-19; Falsely Attacking Long-Term Government Partners Is Unhelpful | <https://www.liberianobserver.com/opinion/the-country-is-pulling-together-to-tackle-covid-19-falsely-attacking-long-term-government-partners-is-unhelpful/> | Liberia | English | Ebola |
| LI-C0125E | June | Daily Observer | KEEP Launches Liberia’s First COVID-19 Book for Early Learners | <https://www.liberianobserver.com/news/keep-launches-liberia-first-coronavirus-book-for-early-learners/> | Liberia | English | Ebola |
| LI-C0126E | June | Daily Observer | LIPACE Launches L$2.2M COVID-19 Care Package | <https://www.liberianobserver.com/news/lipace-launches-l2-2m-covid-19-care-package/> | Liberia | English | Ebola |
| LI-C0129E | June | Daily Observer | ‘No Promotion for Students Post COVID-19’ | <https://www.liberianobserver.com/news/no-promotion-for-students-post-covid-19/> | Liberia | English | Ebola |
| LI-C0130E | June | Daily Observer | Journalists Trained in Gender Sensitive Reporting on COVID-19 and SGBV in Liberia | <https://www.liberianobserver.com/news/spotlight-initiative-supports-media-training-on-gender-sensitive-reporting-on-covid-19-and-sgbv-in-liberia/> | Liberia | English | Ebola |
| LI-C0145E | May | Daily Observer | DKT Liberia Innovates Actions to Mitigate COVID-19 Impact On Access To Family Planning | <https://www.liberianobserver.com/news/dkt-liberia-innovates-actions-to-mitigate-covid-19-impact-on-access-to-family-planning/> | Liberia | English | Ebola |
| LI-C0146E | May | Daily Observer | ActionAid Liberia’s Covid-19 Donations to Boost Operations and Communications for Montserrado EOC Call Center | <https://www.liberianobserver.com/columns/health/actionaid-liberias-covid-19-donations-to-boost-operations-and-communications-for-montserrado-eoc-call-center/> | Liberia | English | Ebola |
| LI-C0156E | May | Daily Observer | Dream Volunteerism Joins Fight against Covid-19 | <https://www.liberianobserver.com/news/dream-volunteerism-joins-fight-against-covid-19/> | Liberia | English | Ebola |
| LI-C0165E | May | Daily Observer | Remain Resilient, Take COVID-19 Preventive Measures Seriously | <https://www.liberianobserver.com/news/remain-resilient-take-covid-19-preventive-measures-seriously/> | Liberia | English | Ebola |
| LI-C0178E | June | Daily Observer | Investing in Young People for COVID-19 Resilience in Liberia | <https://www.liberianobserver.com/opinion/investing-in-young-people-for-covid-19-resilience-in-liberia/> | Liberia | English | Ebola |
| LI-C0533E | March | Daily Observer | ‘No WASCE Until COVID-19 Ceases’ | <https://www.liberianobserver.com/news/no-wasce-until-covid-19-ceases/> | Liberia | English | Ebola |
| LI-C0570E | March | Daily Observer | During COVID-19 Crisis, While All Schools and Universities Are Closed, Orange Liberia Gives Free Access to Educational Content | <https://www.liberianobserver.com/news/during-covid-19-crisis-while-all-schools-and-universities-are-closed-orange-liberia-gives-free-access-to-educational-content/> | Liberia | English | Ebola |
| LI-C0559E | March | Daily Observer | WFP Liberia Completes Installation of Video Conferencing System at NPHIL | <https://www.liberianobserver.com/news/wfp-liberia-completes-installation-of-video-conferencing-system/> | Liberia | English | Ebola |
| LI-C0600E | April | Daily Observer | Rep. Samah Distributes 59 Hand-Washing Buckets to Constituents | <https://www.liberianobserver.com/news/rep-samah-distubts-59-hand-washing-buckets-to-constituents/> | Liberia | English | Ebola |
| LI-C0604E | April | Daily Observer | Rescue Alternatives Wants GoL Protect Detainees | <https://www.liberianobserver.com/news/rescue-alternatives-wants-gol-protect-detainees/> | Liberia | English | Ebola |
| LI-C0610E | April | Daily Observer | MOE Launches “Teaching By Radio” Program | <https://www.liberianobserver.com/news/moe-launches-teaching-by-radio-program/> | Liberia | English | Ebola |
| LI-C0611E | April | Daily Observer | St. Kizito Parish Identifies with Three Vulnerable Communities | <https://www.liberianobserver.com/news/st-kizito-parish-identifies-with-three-vulnerable-communities/> | Liberia | English | Ebola |
| LI-C0613E | April | Daily Observer | How Are Farmers Doing in the Midst of COVID-19? | <https://www.liberianobserver.com/news/how-are-farmers-doing-in-the-midst-of-covid-19/> | Liberia | English | Ebola |
| LI-C0631E | April | Daily Observer | Liberia: Mixed Reactions to Prospects of Lockdown | <https://www.liberianobserver.com/news/liberia-mixed-reactions-to-prospects-of-lockdown/> | Liberia | English | Ebola |
| LI-C0687E | April | Daily Observer | COVID-19 Scare Leaves Saclepea Health Center Deserted | <https://www.liberianobserver.com/news/covid-19-scare-leaves-saclepea-health-center-deserted/> | Liberia | English | Ebola |
| LI-C0697E | April | Daily Observer | Comenius USA Identifies With Liberian Students, amid COVID-19 Fright | <https://www.liberianobserver.com/news/education-comenius-usa-identifies-with-liberian-students-amid-covid-19-fright/> | Liberia | English | Ebola |
| LI-C0706E | April | Daily Observer | One Child Joins COVID-19 Fight | <https://www.liberianobserver.com/news/one-child-joins-covid-19-fight/> | Liberia | English | Ebola |
| LI-C0707E | April | Daily Observer | Preventing and Responding to Sexual and Gender Based Violence During COVID-19 | <https://www.liberianobserver.com/opinion/preventing-and-responding-to-sexual-and-gender-based-violence-during-covid-19/> | Liberia | English | Ebola |
| LI-C0738E | May | Daily Observer | UN Women Supports LNP to Conduct Awareness Campaigns on COVID-19, SGBV | <https://www.liberianobserver.com/news/un-women-supports-lnp-to-conduct-awareness-campaigns-on-covid-19-sgbv/> | Liberia | English | Ebola |
| LI-C0761E | May | Daily Observer | Varney Taylor Foundation Continues Assistance to Medical Centers Amid COVID-19 | <https://www.liberianobserver.com/news/varney-taylor-foundation-continues-assistance-to-medical-centers-amid-covid-19/> | Liberia | English | Ebola |
| LI-C0765E | May | Daily Observer | UN Women Provides Protective Materials for Women, Girls | <https://www.liberianobserver.com/news/un-women-provides-protective-materials-for-women-girls/> | Liberia | English | Ebola |
| LI-C0775E | May | Daily Observer | Is Gov’t Ignoring Risk of Spread of COVID-19 in Prisons? | <https://www.liberianobserver.com/news/is-govt-ignoring-risk-of-spread-of-covid-19-in-prisons/> | Liberia | English | Ebola |
| LI-C0785E | June | Daily Observer | Street Child Assesses COVID-19 Impact on Communities | <https://www.liberianobserver.com/news/street-child-assesses-covid-19-impact-on-communities/> | Liberia | English | Ebola |
| LI-C0791E | June | Daily Observer | ICampus Online Forum Discusses COVID-19’s Impact on Education Sector | <https://www.liberianobserver.com/news/icampus-online-forum-discusses-covid-19s-impact-on-education-sector/> | Liberia | English | Ebola |
| LI-C0793E | June | Daily Observer | Liberia Joins Other Nations to Celebrate World Day Against Child Labour | <https://www.liberianobserver.com/news/liberia-joins-other-nations-to-celebrate-world-day-against-child-labour/> | Liberia | English | Ebola |
| LI-C0805E | March | Daily Observer | Corona Virus: An Ounce of Prevention Is Better Than an Ounce of Cure | <https://www.liberianobserver.com/opinion/corona-virus-an-ounce-of-prevention-is-better-than-an-ounce-of-cure/> | Liberia | English | Ebola |
| LI-C0809E | March | Daily Observer | All Schools Ordered Closed for One Week, As Contact Tracing Begins | <https://www.liberianobserver.com/news/all-schools-ordered-closed-for-one-week-as-contact-tracing-begins/> | Liberia | English | Ebola |
| NA-C0012E | June | Vanguard | COVID-19: Don cautions FG on reopening of schools | <https://www.vanguardngr.com/2020/06/covid-19-don-cautions-fg-on-reopening-of-schools/> | Nigeria | English | Ebola |
| NA-C0016E | June | Vanguard | COVID-19: Parents write minister over inclusion of children with special needs in e-learning | <https://www.vanguardngr.com/2020/06/covid-19-parents-write-open-letter-to-minister-over-inclusion-of-children-with-special-needs-on-e-learning/> | Nigeria | English | Ebola |
| NA-C0025E | June | Vanguard | COVID-19: States, Nigerians not cooperating on testing, FG raises alarm | <https://www.vanguardngr.com/2020/06/covid-19-states-nigerians-not-cooperating-on-testing-fg-raises-alarm/> | Nigeria | English | Ebola |
| NA-  C0029E | June | Vanguard | As Ikpeazu tests positive for Covid-19, Anambra records 80 rape cases during lockdown | <https://www.vanguardngr.com/2020/06/as-ikpeazu-tests-positive-for-covid-19-anambra-records-80-rape-cases-during-lockdown/> | Nigeria | English | Ebola |
| NA-C0054E | June | Vanguard | COVID-19: We can only reopen schools on medical advice — Emeka Nwajiuba | <https://www.vanguardngr.com/2020/06/covid-19-we-can-only-reopen-schools-on-medical-advice-emeka-nwajiuba/> | Nigeria | English | Ebola |
| NA-C0063E | June | Vanguard | Ignoring other diseases over COVID-19, very dangerous ― Dr Ekpenyong | <https://www.vanguardngr.com/2020/06/ignoring-other-diseases-over-covid-19-very-dangerous-%E2%80%95-dr-ekpenyong-2/> | Nigeria | English | Ebola |
| NA-C0074E | June | Vanguard | COVID-19: EU, WFP to transport 52 metric tons of medical equipment in Nigeria | <https://www.vanguardngr.com/2020/06/covid-19-eu-wfp-to-transport-52-metric-tons-of-medical-equipment-in-nigeria/> | Nigeria | English | Ebola |
| NA-C0078E | June | Vanguard | COVID-19: 13 million Nigerians may lose jobs — WFP | <https://www.vanguardngr.com/2020/06/covid-19-13-million-nigerians-may-lose-jobs-wfp/> | Nigeria | English | Ebola |
| NA-C0172E | May | Punch | COVID-19: 28 Almajirai test positive in Kano | <https://punchng.com/covid-19-28-almajirai-test-positive-in-kano/> | Nigeria | English | Ebola |
| NA-C0217E | May | Punch | COVID-19: Gombe discharges 44 Almajirai after 14 days quarantine | <https://punchng.com/covid-19-gombe-discharges-44-almajirai-after-14-days-quarantine/> | Nigeria | English | Ebola |
| NA-C0241E | May | Vanguard | As Lagos battles COVID-19, routine immunisation for babies suffers neglect | <https://punchng.com/as-lagos-battles-covid-19-routine-immunisation-for-babies-suffers-neglect/> | Nigeria | English | Ebola |
| NA-C0258E | May | Punch | COVID-19: I went through hell, says infected pregnant nurse | <https://punchng.com/covid-19-i-went-through-hell-says-infected-pregnant-nurse/> | Nigeria | English | Ebola |
| NA-C0478E | May | Punch | COVID-19: 950 Nigerian children may die daily if… – UNICEF | <https://punchng.com/covid-19-950-nigerian-children-may-die-daily-if-unicef/> | Nigeria | English | Ebola |
| NA-C0497E | May | Vanguard | Mitigating impact of COVID-19 on students with disabilities | <https://punchng.com/mitigating-impact-of-covid-19-on-students-with-disabilities/> | Nigeria | English | Ebola |
| NA-C0833E | April | Vanguard | Oxygen, not ventilators: experts warn of shortages in  poorer virus-threatened nations | <https://www.vanguardngr.com/2020/04/oxygen-not-ventilators-experts-warn-of-shortages-in-poorer-virus-threatened-nations/> | Nigeria | English | Ebola |
| NA-C0921E | April | Vanguard | Presidency lists 50 measures FG has taken to combat coronavirus | <https://www.vanguardngr.com/2020/04/presidency-lists-50-measures-fg-has-taken-to-combat-coronavirus/> | Nigeria | English | Ebola |
| NA-C0953E | March | Punch | COVID-19: Kebbi orders schools’ closure | <https://punchng.com/covid-19-kebbi-orders-schools-closure/> | Nigeria | English | Ebola |
| NA-C1021E | June | Punch | Sexual, domestic abuses worsen COVID-19 trauma for girls, women during lockdown | <https://punchng.com/sexual-domestic-abuses-worsen-covid-19-trauma-for-girls-women-during-lockdown/> | Nigeria | English | Ebola |
| NA-C1074E | June | Punch | COVID-19: Technical challenges rob pupils of virtual classes’ blessings | <https://punchng.com/covid-19-technical-challenges-rob-pupils-of-virtual-classes-blessings/> | Nigeria | English | Ebola |
| NA-C1122E | June | Punch | Government must ensure more pupils don’t drop out after COVID-19 – Don, Dele-Ajayi | <https://punchng.com/government-must-ensure-more-pupils-dont-drop-out-after-covid-19-don-dele-ajayi/> | Nigeria | English | Ebola |
| NA-C1144E | June | Vanguard | Lagos plans education radio station over COVID-19 | <https://punchng.com/lagos-plans-education-radio-station-over-covid-19/> | Nigeria | English | Ebola |
| NR-C0001F | May | Le Sahel | Reprise Des Cours Dans Le Contexte De La COVID-19 : Relever Le Défi Du Respect Du Programme Et Des Mesures De Prévention Édictées | <http://www.lesahel.org/index.php/2020/05/29/reprise-des-cours-dans-le-contexte-de-la-covid-19-relever-le-defi-du-respect-du-programme-et-des-mesures-de-prevention-edictees/> | Niger | French | Non-Ebola |
| NR-C0004F | June | Le Sahel | Réunion Virtuelle Du Comité Régional De Pilotage Du Projet SWEDD : Assurer La Continuité Des Services Du Projet SWEDD Malgré La Pandémie À Coronavirus | <http://www.lesahel.org/reunion-virtuelle-du-comite-regional-de-pilotage-du-projet-swedd-assurer-la-continuite-des-services-du-projet-swedd-malgre-la-pandemie-a-coronavirus/> | Niger | French | Non-Ebola |
| NR-C0006F | May | Le Sahel | Le Ministre En Charge De L’Enseignement PriMayre Visite Des Établissements Scolaires À Niamey : «Ce Que J’ai Constaté Me Donne L’espoir Que L’école Ne Sera Pas Un Lieu De Contamination De La Maladie À Coronavirus», Déclare M. Daouda Mamadou Marthé | [http://www.lesahel.org/le-ministre-en-charge-de-lenseignement-primaire-visite-des-etablissements-scolaires-a-niamey-ce-que-jai-constate-me-donne-lespoir-que-lecole-ne-sera-pa/#](http://www.lesahel.org/le-ministre-en-charge-de-lenseignement-primaire-visite-des-etablissements-scolaires-a-niamey-ce-que-jai-constate-me-donne-lespoir-que-lecole-ne-sera-pa/) | Niger | French | Non-Ebola |
| NR-C0017F | April | Le Sahel | Lutte Contre Le Coronavirus : Le Président De La République Gracie 1540 Détenus Dont M. Hama Amadou | <http://www.lesahel.org/lutte-contre-le-coronavirus-le-president-de-la-republique-gracie-1540-detenus-dont-m-hama-amadou-7/> | Niger | French | Non-Ebola |
| NR-C0032F | May | A Niamey | (COVID-19) Des partenaires viennent au secours de plus de 27.000 enfants victimes de malnutrition au Niger | <http://news.aniamey.com/h/97929.html> | Niger | French | Non-Ebola |
| NR-C0044F | April | A Niamey | Le chef de l’ONU met en garde contre la flambée de violence domestique liée au confinement en réponse au COVID-19 | <http://news.aniamey.com/h/97083.html> | Niger | French | Non-Ebola |
| NR-C0049F | June | A Niamey | (COVID-19) Le chef de l’OMS encourage les femmes infectées à continuer d’allaiter | <http://news.aniamey.com/h/98220.html> | Niger | French | Non-Ebola |
| NR-C0063F | May | Le Sahel | Protection Sociale Des Femmes En Période De Pandémie Du Covid-19 : Le Genre Doit Être Pris En Compte Dans La Réponse Face Au COVID-19 | <http://www.lesahel.org/protection-sociale-des-femmes-en-periode-de-pandemie-du-covid-19-le-genre-doit-etre-pris-en-compte-dans-la-reponse-face-au-covid-19/> | Niger | French | Non-Ebola |
| NR-C0071F | June | A Niamey | COVID-19 : Le Médiateur de la République en campagne de sensibilisation dans les écoles des régions de Dosso et Maradi | <http://news.aniamey.com/h/98241.html> | Niger | French | Non-Ebola |
| NR-C0094F | June | Le Sahel | Lancement Officiel Du Concours Média 2020 : «Pour Chaque Enfant Un Champion : La Crise De Covid 19 Est Une Crise Des Droits De L’enfant», Thème De L’édition | <http://www.lesahel.org/index.php/2020/06/10/lancement-officiel-du-concours-media-2020-pour-chaque-enfant-un-champion-la-crise-de-covid-19-est-une-crise-des-droits-de-lenfant-theme-de-ledition/> | Niger | French | Non-Ebola |
| NR-C0096F | June | Le Sahel | Tattali Iyali Foundation: Constant Support To Vulnerable Groups During Ramadan And In The Context Of Resilience To Covid-19 | <http://www.lesahel.org/fondation-tattali-iyali-un-accompagnement-constant-aux-couches-vulnerables-pendant-le-ramadan-et-dans-le-cadre-de-la-resilience-au-covid-19/> | Niger | French | Non-Ebola |
| NR-C0155F | April | A Niamey | Covid-19: l’endiguement encore possible en Afrique, selon l’OMS | <http://news.aniamey.com/h/97137.html> | Niger | French | Non-Ebola |
| NR-C0178F | June | A Niamey | COVID-19 pandemic accentuates humanitarian needs in the Sahel, UN says | <http://news.aniamey.com/h/98332.html> | Niger | French | Non-Ebola |
| NR-C0206F | June | A Niamey | La pandémie de COVID-19 accentue les besoins humanitaires au Sahel, selon l’ONU | <http://news.aniamey.com/h/98332.html> | Niger | French | Non-Ebola |
| NR-C0209F | June | A Niamey | L’OMS inquiète de l’impact de COVID-19 sur les femmes et les filles en Afrique | <http://news.aniamey.com/h/98322.html> | Niger | French | Non-Ebola |
| NR-C0233F | June | A Niamey | Lutte contre la COVID19 en milieu carcéral : La CNDH effectue un monitoring dans les prisons et procède à la remise de dons en kits sanitaires | <http://news.aniamey.com/h/98396.html> | Niger | French | Non-Ebola |
| NR-C0258F | June | A Niamey | Lutte Contre La COVID19 En Milieu Carcéral : La CNDH Effectue Un Monitoring Dans Les Prisons Et Procède À La Remise De Dons En Kits Sanitaires | <http://www.lesahel.org/lutte-contre-la-covid19-en-milieu-carceral-la-cndh-effectue-un-monitoring-dans-les-prisons-et-procede-a-la-remise-de-dons-en-kits-sanitaires/> | Niger | French | Non-Ebola |
| SL-C0045E | May | Awoko | FCC, partners targeting 350,000 vulnerable in COVID-19 fight | <https://awokonewspaper.com/fcc-partners-targeting-350000-vulnerable-in-covid-19-fight/> | Sierra Leone | English | Ebola |
| SL-C0067E | May | Awoko | Salone to witness highest excess child mortality rates during COVID-19 –John Hopkins | <https://awokonewspaper.com/salone-to-witness-highest-excess-child-mortality-rates-during-covid-19-john-hopkins/> | Sierra Leone | English | Ebola |
| SL-C0118E | April | Awoko | Salone among 38 countries to benefit from GPE US$250m COVID-19 educational support | <https://awokonewspaper.com/salone-among-38-countries-to-benefit-from-gpe-us250m-covid-19-educational-support/> | Sierra Leone | English | Ebola |
| SL-C0130E | March | Awoko | COVID-19: Advocaid calls for Release pre-trial and vulnerable detainees | <https://awokonewspaper.com/covid-19-advocaid-calls-for-release-pre-trial-and-vulnerable-detainees/> | Sierra Leone | English | Ebola |
| SL-C0139E | June | Awoko | Antonio Rudiger Foundation Sierra Leone Donates, Feeds Over 200 Children In Kroo Bay Community | <https://awokonewspaper.com/antonio-rudiger-foundation-sierra-leone-donates-feeds-over-200-children-in-kroo-bay-community/> | Sierra Leone | English | Ebola |
| SL-C0140E | June | Awoko | UNFPA distributes mama-baby packs to pregnant women | <https://awokonewspaper.com/unfpa-distributes-mama-baby-packs-to-pregnant-women/> | Sierra Leone | English | Ebola |
| SL-C0174E | June | Awoko | UMC Women donate food to pregnant women, lactating mothers | <https://awokonewspaper.com/umc-women-donate-food-to-pregnant-women-lactating-mothers/> | Sierra Leone | English | Ebola |
| SL-C0195E | May | Awoko | Antenatal visits increase at Kingharman road Hospital | <https://awokonewspaper.com/antenatal-visits-increase-at-kingharman-road-hospital/> | Sierra Leone | English | Ebola |
| SL-C0205E | May | Awoko | Social Workers feed homeless, mentally challenged people on Sundays | <https://awokonewspaper.com/social-workers-feed-homeless-mentally-challenged-people-on-sundays/> | Sierra Leone | English | Ebola |
| SL-C0208E | May | Awoko | Lactating mothers and pregnant women abandon health facilities in Tonkolili | <https://awokonewspaper.com/lactating-mothers-and-pregnant-women-abandon-health-facilities-in-tonkolili/> | Sierra Leone | English | Ebola |
| SL-C0210E | May | Awoko | CUAMM support health sector | <https://awokonewspaper.com/cuamm-support-health-sector/> | Sierra Leone | English | Ebola |
| SL-C0214E | May | Awoko | School closures give rise to more children selling on the street | <https://awokonewspaper.com/school-closures-give-rise-to-more-children-selling-on-the-street/> | Sierra Leone | English | Ebola |
| SL-C0235E | May | Awoko | Amnesty blames Govt. for prison riot due to delay in protecting their health right | <https://awokonewspaper.com/amnesty-blames-govt-for-prison-riot-due-to-delay-in-protecting-their-health-right/> | Sierra Leone | English | Ebola |
| SL-C0242E | May | Awoko | SWSG, Beauties with brains partnership donate to girls in Kroobay | <https://awokonewspaper.com/swsg-beauties-with-brains-partnership-donate-to-girls-in-kroobay/> | Sierra Leone | English | Ebola |
| SL-C0335E | June | Awoko | Global Partnership for Education increases COVID-19 emergency fund by US$250m | <https://awokonewspaper.com/global-partnership-for-education-increases-covid-19-emergency-fund-by-us250m/> | Sierra Leone | English | Ebola |
| SL-C0369E | April | Sierra Leone Telegraph | Dangers of covid-19 – is government of Sierra Leone capable and ready to contain its spread? | <https://www.thesierraleonetelegraph.com/dangers-of-covid-19-is-government-of-sierra-leone-capable-and-ready-to-contain-its-spread/> | Sierra Leone | English | Ebola |
| SL-C0459E | April | Sierra Leone Telegraph | Corona lockdowns expose young girls to unintended risks in Sierra Leone | <https://www.thesierraleonetelegraph.com/corona-lockdowns-expose-young-girls-to-unintended-risks-in-sierra-leone/> | Sierra Leone | English | Ebola |
| SL-C0471E | June | Sierra Leone Telegraph | Acute water shortage at Pademba Road prisons as number of covid-19 cases rises | <https://www.thesierraleonetelegraph.com/acute-water-shortage-at-pademba-road-prisons-as-number-of-covid-19-cases-rises/> | Sierra Leone | English | Ebola |
| SL-C0472E | June | Sierra Leone Telegraph | COVID-19 jeopardises progress in protecting women and girls from violence and harmful practices | <https://www.thesierraleonetelegraph.com/covid-19-jeopardises-progress-in-protecting-women-and-girls-from-violence-and-harmful-practices/> | Sierra Leone | English | Ebola |
| SL-C0485E | June | Awoko | Chinese Ambassador donates to First Lady | <https://awokonewspaper.com/chinese-ambassador-donates-to-first-lady/> | Sierra Leone | English | Ebola |
| SL-C0517E | June | Awoko | MBSSE gets GPE’s approval for US$7m COVID-19 educational response | <https://awokonewspaper.com/mbsse-gets-gpes-approval-for-us7m-covid-19-educational-response/> | Sierra Leone | English | Ebola |
| SL-C0521E | June | Awoko | Ministry of Social Welfare receives 32 motorbikes to fight COVID-19 | <https://awokonewspaper.com/ministry-of-social-welfare-receives-32-motorbikes-to-fight-covid-19/> | Sierra Leone | English | Ebola |

### S4 Table. Country-Specific Table on Frequency of Impacts (From January 1 to June 30, 2020).

| Themes | Sub-themes | Countries | | | | | |  |
| --- | --- | --- | --- | --- | --- | --- | --- | --- |
|  |  | Liberia | Sierra Leone | Guinea | Nigeria | Niger | Ghana | Total # of excerpts |
|  |  | High Ebola | High Ebola | Moderate Ebola | Moderate Ebola | No  Ebola | No Ebola |  |
| Mothers and Children | 1. Disruptions in Education | 19 | 3 | 1 | 26 | 2 | 6 | 57 |
|  | 2. Reduction in Care Seeking Behaviors and Access to Health Care | 7 | 9 | 6 | 10 | 0 | 1 | 33 |
|  | 3. Food and Economic Insecurity | 5 | 1 | 0 | 0 | 5 | 1 | 12 |
|  | 4. Sexual Abuse and Other Forms of Domestic Violence | 8 | 4 | 2 | 7 | 2 | 4 | 27 |
| Total |  |  |  |  |  |  |  | **129** |
| Prisoners and Prison Staff | 1. Overcrowded Prisons and panic concerning COVID-19 | 2 | 1 | 13 | 0 | 0 | 0 | 16 |
|  | 2. Lack of Food and Water | 0 | 4 | 1 | 0 | 0 | 0 | 5 |
| Total |  |  |  |  |  |  |  | **21** |
| Informal Sector Workers and Poor Populations | 1. Worsened Food Insecurity | 5 | 0 | 1 | 0 | 1 | 9 | 16 |
|  | 2. Inability to Farm/Sell Products | 2 | 0 | 4 | 0 | 0 | 2 | 8 |
| Total |  |  |  |  |  |  |  | **24** |
| Elderly and Adult Disabled Populations | 1. Poverty, Stigma, and Lack of Access to Assistance | 0 | 0 | 0 | 0 | 1 | 0 | 1 |
| Total |  |  |  |  |  |  |  | **1** |
| Total # of Excerpts |  | **48** | **22** | **28** | **43** | **11** | **23** | **175** |

**
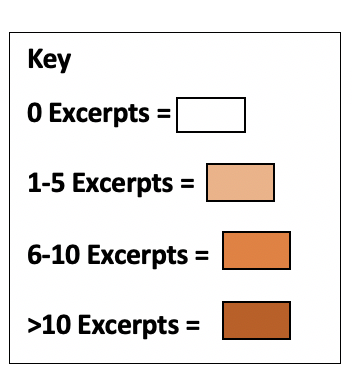
**

### S5 Table. Solutions by Impacts (From January 1 to June 30, 2020).

| **Population** | **Effect** | **Solutions** | | | | | |
| --- | --- | --- | --- | --- | --- | --- | --- |
|  |  | Local and Foreign Donations | Technical Assistance to Improve Programming | Technological Innovation and Information for Children | Release of Detainees and Protection for Prisoners | Maintaining Access to Coronavirus Related Prevention and Health Services | Total # of Excerpts |
| **Mothers and Children** | 1. Disruptions in Education | 6 | 1 | 17 | 0 | 0 | 24 |
|  | 2. Reduction in Care Seeking Behaviors and Access to Health Care | 12 | 1 | 0 | 0 | 0 | 13 |
|  | 3. Food and Economic Insecurity | 16 | 1 | 0 | 0 | 0 | 17 |
|  | 4. Sexual abuse and other forms of domestic violence | 2 | 0 | 0 | 0 | 0 | 2 |
| **Prisoners and Prison Staff** | 5. Overcrowded Prisons and Panic Concerning COVID-19 | 0 | 0 | 0 | 10 | 6 | 16 |
|  | 6. Lack of Food and Water | 3 | 0 | 0 | 1 | 8 | 12 |
| **Informal Sector Workers and Poor Populations** | 7. Worsened Food Insecurity | 13 | 2 | 0 | 0 | 0 | 15 |
|  | 8. Inability to Farm/Sell Products | 0 | 5 | 0 | 0 | 0 | 5 |
| **Elderly and Adult Disabled Population** | 9. Poverty, Stigma, and Lack of Access to Assistance | 6 | 0 | 0 | 0 | 0 | 6 |
| **Total # of Excerpts** |  | 58 | 10 | 17 | 11 | 14 | 110 |

**
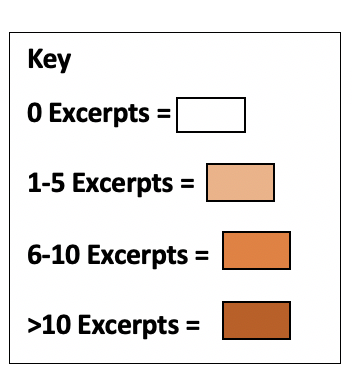
**
